# Recent Advances in Photocatalyst‐Driven Protein Labeling and Proximity Mapping

**DOI:** 10.1002/tcr.70180

**Published:** 2026-06-01

**Authors:** Shinichi Sato, Kazuki Miura, Juki Nakao, Hiroyuki Nakamura

**Affiliations:** ^1^ Frontier Research Institute for Interdisciplinary Sciences Tohoku University Sendai Miyagi Japan; ^2^ Graduate School of Life Sciences Tohoku University Sendai Miyagi Japan; ^3^ Laboratory for Chemistry and Life Science Institute of Integrated Research Institute of Science Tokyo Yokohama Kanagawa Japan

**Keywords:** energy transfer, photocatalyst, protein chemical labeling, proximity labeling, single‐electron transfer

## Abstract

Photocatalysis has emerged as a powerful strategy for controlling chemical reactivity with light, offering unique opportunities for spatial and temporal regulation. While visible‐light photocatalysis was originally developed in the context of small‐molecule synthesis, recent years have witnessed its rapid expansion into the selective modification of peptides and proteins under biologically compatible conditions. When photocatalysts are localized through ligands, antibodies, nanomaterials, or genetic fusion, photochemical reactivity becomes confined to defined molecular neighborhoods, giving rise to photocatalytic proximity labeling as a distinct chemical approach for probing biomolecular interactions. This review summarizes advances in photocatalyst‐enabled protein modification and proximity labeling reported up to 2025. We highlight how diverse photochemical mechanisms—including single‐electron transfer, energy transfer, and the generation of short‐lived reactive intermediates such as radicals, carbenes, nitrenes, and singlet oxygen—have been harnessed across experimental regimes ranging from purified proteins and solid‐supported platforms to living cells, tissues, and in vivo systems. Collectively, these developments establish photocatalysis as a versatile chemical framework for rational design of proximity labeling tools with tunable spatial resolution, enabling spatial encoding in biological systems and the interrogation of protein interactions within complex biological environments.

## Graphical abstract

Photocatalyst‐driven protein labeling and proximity mapping have rapidly advanced as powerful strategies for spatiotemporal control in complex biological environments. This review integrates recent developments across single‐electron transfer and energy transfer‐based mechanisms and highlights how catalyst design, reactive intermediates, and diffusion governs labeling radius and selectivity. A unified framework is presented to guide the rational design and application of these systems from in vitro to in vivo contexts.

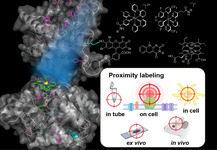

## 1 Introduction

Photochemistry has long provided a means to control chemical reactivity through external light input, offering unique advantages in spatial and temporal regulation. Over the past decade, the development of visible‐light photocatalysis has substantially expanded the scope of synthetic chemistry, enabling reactions to proceed under mild conditions and allowing controlled access to radical and excited‐state reactivity. Although these advances were initially established in small‐molecule synthesis, photocatalytic concepts have more recently been extended to the selective modification of peptides and proteins. In such contexts, light serves as an effective trigger to access reaction pathways that are otherwise difficult to realize under aqueous and biologically compatible conditions. Importantly, the application of photocatalysis is no longer limited to reactions conducted in flasks, but has begun to extend into living cells and biological tissues, where chemical reactivity can be regulated directly within complex biological environments.

The adaptation of photocatalytic reactions to peptide and protein modification has opened new directions at the interface of chemistry and biology. Visible‐ or red‐light‐driven photocatalysis enables residue‐selective transformations without the need for harsh reagents, thereby providing practical tools for bioconjugation, late‐stage functionalization, and chemical interrogation of protein structure and function. When photocatalysts are localized through small‐molecule ligands, antibodies, nanomaterials, or genetic fusion, the resulting photochemical reactivity becomes spatially restricted. Under these conditions, photocatalytic protein modification acquires a proximity‐dependent character, in which covalent labeling preferentially occurs within a defined molecular neighborhood. This realization has led to the emergence of photocatalytic proximity labeling as a distinct methodological concept.

In parallel, proximity labeling has become an important strategy for studying biomolecular interactions in their native context. Protein–protein interactions (PPIs), transient encounter complexes, and spatially organized molecular assemblies play central roles in cellular signaling and regulation, yet are often challenging to capture using conventional biochemical approaches. Proximity labeling addresses this challenge by converting spatial proximity into covalent modifications that can be analyzed by mass spectrometry or imaging, enabling systematic characterization of local protein environments.

Among existing approaches, enzyme‐based proximity labeling methods such as APEX, BioID, and TurboID have been widely adopted. APEX employs an engineered peroxidase to generate phenoxyl radicals from phenolic substrates in the presence of hydrogen peroxide, resulting in rapid labeling of nearby proteins. BioID and TurboID are based on promiscuous biotin ligases that produce reactive biotin–AMP intermediates, which label proximal lysine (Lys) residues over longer time scales. These methods offer the advantage of genetic targetability and have proven effective for interactome mapping in living cells and organisms.

At the same time, enzymatic proximity labeling inherently depends on catalytic turnover and diffusion of reactive intermediates. As a consequence, labeling radii are often relatively broad, and temporal resolution is constrained by enzyme kinetics and substrate availability. Prolonged labeling periods may also lead to elevated background signals. In addition, the use of exogenous cofactors or extended incubation under biotin‐rich conditions can perturb cellular environments, complicating the analysis of weak or transient interactions.

Photocatalytic proximity labeling represents a mechanistically distinct and complementary approach. In these systems, light functions as an external switch that activates photocatalysts only during defined irradiation periods, providing intrinsic temporal control and minimizing background labeling in the absence of light. The reactive intermediates generated through photocatalysis—including radicals, carbenes, nitrenes, and singlet oxygen (^1^O_2_)—exhibit lifetimes and diffusion ranges that are governed by photophysical properties rather than enzymatic amplification. This feature allows spatial resolution to be tuned through reaction design and photocatalyst selection.

A number of comprehensive review articles have surveyed the broader landscape of photocatalysis in biological research, including visible‐light‐enabled manipulation of biomacromolecules, photocatalyst structures for protein modification, catalytic strategies for bioconjugation, and emerging therapeutic challenges associated with catalytic chemistry [1, 2, 3, 4, 5, 6, 7]. In this review, we summarize recent developments in photocatalyst‐enabled protein modification and proximity labeling strategies reported up to 2025, with particular emphasis on how photochemical reactivity is harnessed and adapted across different biological contexts. Rather than providing an exhaustive survey of photocatalysis in biology, we focus on systems in which photocatalysts play an active and spatially defined role in protein modification, and on how these platforms have evolved from fundamental chemical concepts into practical tools for probing protein interactions and molecular microenvironments in complex biological settings. By comparing photocatalytic strategies with direct photoexcitation methods and enzyme‐based proximity labeling techniques, we aim to clarify their respective advantages and limitations and to provide an organized perspective on how photochemical approaches can be applied to study biomolecular interactions across different levels of biological complexity.

## 2 Photocatalysts for Peptide and Protein Modification

Light‐mediated modification of peptides and proteins offers high spatiotemporal resolution, representing a significant advantage for applications in chemical biology. Historically, numerous studies have reported the use of highly reactive intermediates generated upon light irradiation of stable precursors for targeted labeling and interaction analysis.

Representative photoreactive groups include aryl azides, diazirines, and benzophenones, which, upon ultraviolet (UV) irradiation, generate nitrenes, carbenes, and ketyl radicals, respectively [8, 9]. Nitrenes are highly electrophilic species and can form covalent bonds non‐selectively with a broad range of amino acid residues, allowing robust target labeling. In contrast, carbenes have a shorter lifetime than nitrenes, and their reactivity is thus more locally confined, resulting in relatively higher selectivity. Ketyl radicals are produced via hydrogen abstraction from surrounding C—H bonds by the triplet excited state of benzophenone, achieving preferential labeling of hydrophobic amino acid residues. Direct excitation strategies generating such reactive intermediates have been widely employed in various applications, including photoaffinity labeling [8, 9].

However, the use of UV light presents several challenges. Endogenous biomolecules, such as DNA bases, flavins, and porphyrins, absorb UV light, potentially causing undesired side reactions and damage to proteins and nucleic acids [10, 11]. To overcome these limitations, recent approaches have focused on visible and near‐infrared (NIR) light, which allow modifications under milder conditions with higher selectivity. In particular, photocatalyst‐mediated reactions are promising, enabling the use of low‐energy light while enhancing control over reaction pathways.

Considering the diversity of developed photocatalysts, peptide and protein modification via photocatalysis can be broadly categorized into two catalytic mechanisms: single‐electron transfer (SET) and energy transfer (EnT). Unlike direct photoexcitation strategies that rely on UV light to generate reactive intermediates from stable precursors, catalytic photochemical approaches enable reactions under milder visible‐light conditions through the mediation of photoredox or photosensitizing catalysts. In this section, we provide an overview of peptide and protein modification strategies based on these two catalytic mechanisms, organizing our discussion by photocatalyst structure and highlighting recent advances in catalyst design and reaction development.

### 2.1 Photocatalysts for Electron Transfer Mechanisms

SET‐based modification involves the generation of radical intermediates through electron transfer reactions between a photoexcited catalyst and either a labeling reagent or the peptide and protein itself. Upon visible‐light excitation, the catalyst acts as a potent oxidant or reductant, generating radical species under mild conditions. Such radical intermediates are particularly useful for introducing functional molecules into specific biomolecules within cellular environments. By exploiting the high reactivity of radicals, site‐selective chemical modification of amino acid residues, which is difficult to achieve with traditional two‐electron bioconjugation, becomes possible.

In SET‐mediated photocatalysis, residues such as tyrosine (Tyr), tryptophan (Trp), histidine (His), methionine (Met), and C‐terminal carboxylic acids serve as primary targets, enabling selective modification of previously inaccessible functional groups. In visible‐light‐induced photoredox reactions, amino acid residues or externally added labeling reagents function as electron donors or acceptors, generating radicals via the SET process, which subsequently form stable covalent bonds. This design provides mild reaction conditions, high site‐selectivity, and excellent functional group tolerance, making it a powerful next‐generation bioconjugation strategy.

Nonetheless, SET‐based modification presents challenges, including potential off‐target reactions from highly reactive residues such as cysteine (Cys) and Lys [12]. Some reports have indicated side reactions, such as protein crosslinking and overoxidation, likely due to the strong oxidizing power of the excited photoredox catalyst [13]. Careful selection of catalysts with appropriate redox properties and reaction conditions is therefore crucial for efficient and reproducible protein modification. The following sections highlight representative examples, illustrating the diversity and potential of SET‐based strategies and providing guidance for selecting optimal photocatalysts and reaction mechanisms based on the intended modification.

#### 2.1.1 Ruthenium Complexes

**FIGURE 1:**
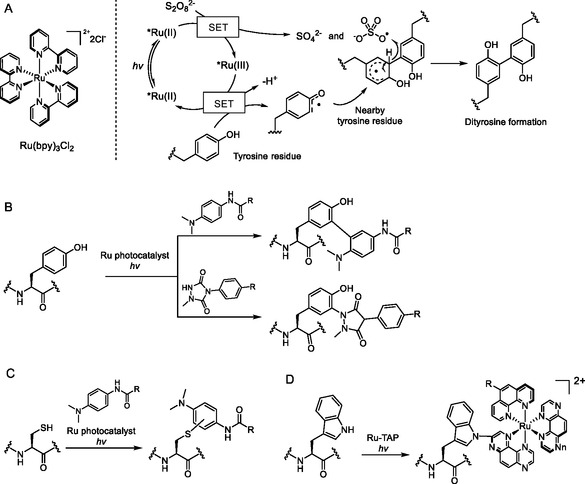
Peptide and protein modification via the SET mechanism using Ru complexes. (A) Structure of Ru complex and Tyr–Tyr cross‐coupling reaction. (B) Tyr modification with dimethylphenylenediamine and 1‐methyl‐4‐arylurazole (MAUra) derivatives. (C) Cys modification with dimethylphenylenediamine derivatives. (D) Trp modification with Ru‐TAP.

Visible‐light‐responsive ruthenium (Ru) complexes, particularly Ru(bpy)_3_ derivatives, serve as robust photoredox catalysts for selective peptide and protein modification due to their stability, visible‐light absorption, and reversible redox properties. In 1999, Fancy and Kodadek demonstrated that visible‐light irradiation of Ru(bpy)_3_Cl_2_ in the presence of ammonium persulfate drove an oxidative catalytic cycle, generating Ru(III) from the excited Ru(II)*. Ru(III) accepted an electron from Tyr residues, forming tyrosyl radicals, which participated in dityrosine formation or crosslinking with nucleophilic side chains, establishing the first example of Ru‐catalyzed SET protein modification (Figure 1A) [14].

Building on this Tyr‐selective platform, efforts have been made to expand and control the selectivity and applicability of Ru‐based photocatalysts through molecular design and strategic combination with labeling reagents. We first demonstrated that Ru(bpy)_3_ photocatalysts preferentially label tyrosine residues in proteins via photoinduced SET mechanisms [15]. This approach established the conceptual framework for proximity‐dependent photoredox labeling, where spatial localization of the catalyst enables selective modification even in complex protein mixtures (detailed applications are described in Section 3.1) (Figure 1B).

Building on this Tyr‐selective platform, Beard and colleagues reported a proximity labeling approach using phenylenediamine as a radical‐trapping reagent. In specific cases where a free Cys residue was located in close proximity to the catalyst, Cys labeling could be achieved, representing a special case rather than a general shift in the inherent Tyr selectivity of Ru photocatalysts [16]. This context‐dependent selectivity shift—where proximity effects override intrinsic residue reactivity trends—demonstrated that Cys labeling could be achieved not through a change in the photocatalyst's fundamental mechanism, but through strategic positioning of the catalyst relative to nucleophilic residues (Figure 1C).

To further control residue selectivity, Chen and colleagues reported a method for Trp–selective photo‐labeling using peptides conjugated with Ru‐TAP complexes. Upon visible‐light irradiation, the excited Ru‐TAP preferentially accepted an electron from the indole ring of proximal Trp residues, generating Trp radical cations. These radical intermediates rapidly recombined or underwent addition reactions with reactive sites on Ru‐TAP, forming covalent bonds with Trp residues. Tyr residues, in contrast, were less prone to SET, and any resulting phenoxyl radicals were readily quenched, preventing covalent bond formation. Thus, both the initial SET event and subsequent reaction steps contributed to the high residue selectivity observed for Trp (Figure 1D) [17].

Mechanism switching between SET and EnT pathways has also been demonstrated with Ru photocatalysts. We showed that several photocatalysts could operate via two distinct mechanisms depending on reaction conditions and catalyst positioning [18, 19]. In many cases, SET and EnT processes can operate in parallel upon photoexcitation:Tyr labeling proceeds via SET, while the EnT‐mediated His labeling proceeds via ^1^O_2_ generation (mechanism and applications are detailed in Section 2.2.1). The observed labeling selectivity—whether tyrosine or histidine predominates—depends on the relative kinetics of these competing pathways. When the photocatalyst structure favors efficient SET or when Tyr residues are positioned in close proximity to the catalyst, tyrosine modification dominates through the SET pathway, enabling localized and highly selective Tyr labeling. Conversely, under conditions where EnT dominates over SET, ^1^O_2_ can be generated as the primary reactive intermediate, which preferentially oxidizes histidine residues. These studies established that photocatalysts function as versatile platforms whose operative mechanism—“localized electron transfer source” versus “^1^O_2_ generator”—can be controlled through rational design of both catalyst structure and spatial positioning, enabling programmable residue selectivity and proximity‐labeling behavior.

#### 2.1.2 Iridium Complexes

Similar to Ru complexes, iridium (Ir)‐based photocatalysts exhibit excellent redox capabilities and high photostability under visible‐light irradiation, enabling site‐selective modification of otherwise challenging amino acid residues. The ability to finely tune redox potentials and excited‐state lifetimes via ligand design is a key advantage of Ir catalysts.

**FIGURE 2:**
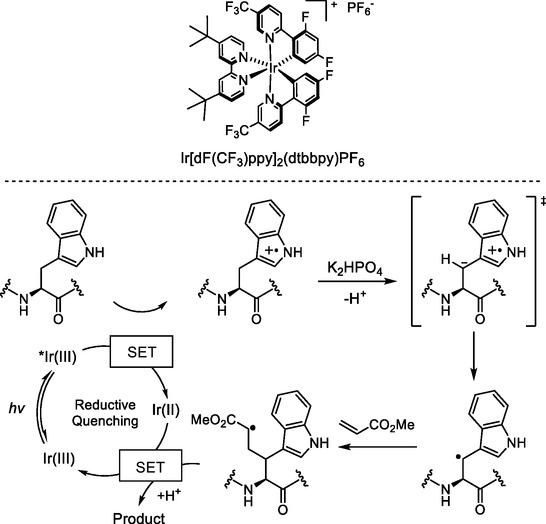
Peptide and protein modification via the SET mechanism using Ir complex. Structure of Ir complex and tryptophan modification with methyl acrylate.

Yu and colleagues reported a method for selective β‐functionalization of Trp residues in peptides and proteins using Ir[dF(CF_3_)ppy]_2_(dtbbpy)(PF_6_) as the photocatalyst. β‐Position modification of Trp had been rarely achieved, and this strategy was notable for exploiting a novel reaction site beyond the indole ring, enabling the introduction of diverse functionalities into biomolecules. Mechanistically, visible‐light irradiation excited Ir(III)*, which underwent SET with the indole nitrogen to generate a Trp radical cation. Subsequent radical rearrangement and deprotonation at the β‐position yielded a relatively stable β‐tryptophan radical (skatolyl radical). This radical reacted with electron‐deficient alkenes, such as methyl acrylate, and upon electron donation from reduced Ir(II) followed by protonation, the β‐functionalized product was formed (Figure 2). Competing reactions, such as Michael addition of lysine or histidine via two‐electron pathways or side reactions involving C‐terminal decarboxylation, could occur depending on the substrate and reaction conditions, highlighting the importance of reaction control [20].

Exploiting the high reactivity of Ir photocatalysts, efforts have been made to expand site‐selective modification to noncanonical amino acids. Bruijin and Roelfes reported selective modification of dehydroalanine (Dha) residues in peptides and proteins via an Ir‐catalyzed SET mechanism. Upon visible‐light irradiation, the excited Ir photocatalyst oxidized radical precursors, such as alkylboron compounds, to generate carbon‐centered radicals. These radicals underwent Giese‐type addition to the electron‐deficient double bond of Dha, forming new C—C bonds. The resulting radicals were stabilized through single‐electron reduction by the reduced catalyst and protonation or hydrogen‐atom transfer, yielding the modified product. This method achieved high site‐selectivity under aqueous and mild conditions by combining the high radical acceptor reactivity of Dha with an external radical‐supply strategy [21].

Furthermore, Dowman and colleagues reported a highly selective method for modifying selenocysteine (Sec) residues using an Ir‐catalyzed SET mechanism. Visible‐light irradiation excited Ir(III)*, which oxidized a phosphorus reagent to generate a reactive species that selectively activated and cleaved the diselenide (Se—Se) bond of Sec residues. Subsequent electron transfer and bond rearrangement led to “diselenide contraction,” forming stable selenoether linkages. Due to the higher redox activity of selenium compared to sulfur and the relative weakness of Se—Se bonds, Cys residues remained unreactive under the same conditions, enabling Sec‐specific functionalization. This approach represented a mild and rapid site‐selective protein modification strategy that leveraged the cooperative action of photoinduced SET and phosphorus reagents [22].

#### 2.1.3 Tin Complexes

Beyond residue selectivity, excitation wavelength has emerged as a critical design parameter, particularly for applications in tissues or in vivo systems, where light penetration and phototoxicity become limiting factors.

From the perspectives of improving biocompatibility and enhancing light penetration into tissues, recent efforts have focused on extending the excitation wavelengths of photocatalysts from the short‐wavelength visible region to the red and NIR regions. Long‐wavelength light exhibits reduced scattering and absorption by biological tissues and lower phototoxicity, making it particularly advantageous for protein modification and proximity labeling in cellular or in vivo settings. In this section, we summarize a representative metal‐based photocatalyst system suitable for amino acid residue modification under red to NIR light irradiation, specifically focusing on tin (Sn) complexes.

**FIGURE 3:**
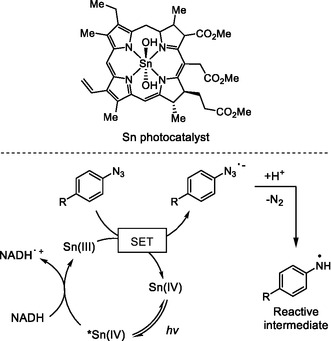
Protein modification via the SET mechanism using Sn complex. Structure of Sn complex and any amino acids modification with aryl azide derivatives.

Buksh and colleagues developed μMap‐Red, a proximity labeling method that combined a Sn–chlorin e6 photocatalyst with aryl azide‐based labeling probes. Sn–chlorin e6 was a chlorophyll‐derived complex that exhibited high molar absorptivity in the red‐light region while maintaining stable redox properties under biological conditions. Mechanistically, red‐light irradiation excited Sn(IV)–chlorin e6, which was subsequently reductively quenched by intracellular reducing agents such as NADH to generate the Sn(III) species. The Sn(III) intermediate displayed strong reducing power and activated nearby aryl azides via single‐electron reduction to generate azide radical anion. This activation led to nitrogen extrusion and formation of a short‐lived aminyl radical or nitrene‐like intermediate. Due to its extremely short diffusion distance, this reactive intermediate selectively formed covalent bonds with amino acid side chains in close proximity to the photocatalyst‐localized protein (Figure 3). Sn–chlorin e6 combines strong red‐light absorption with sufficient reducing potential, enabling enhanced tissue penetration and reduced phototoxicity compared to conventional methods using UV or blue light [23].

#### 2.1.4 Flavin Derivatives

**FIGURE 4:**
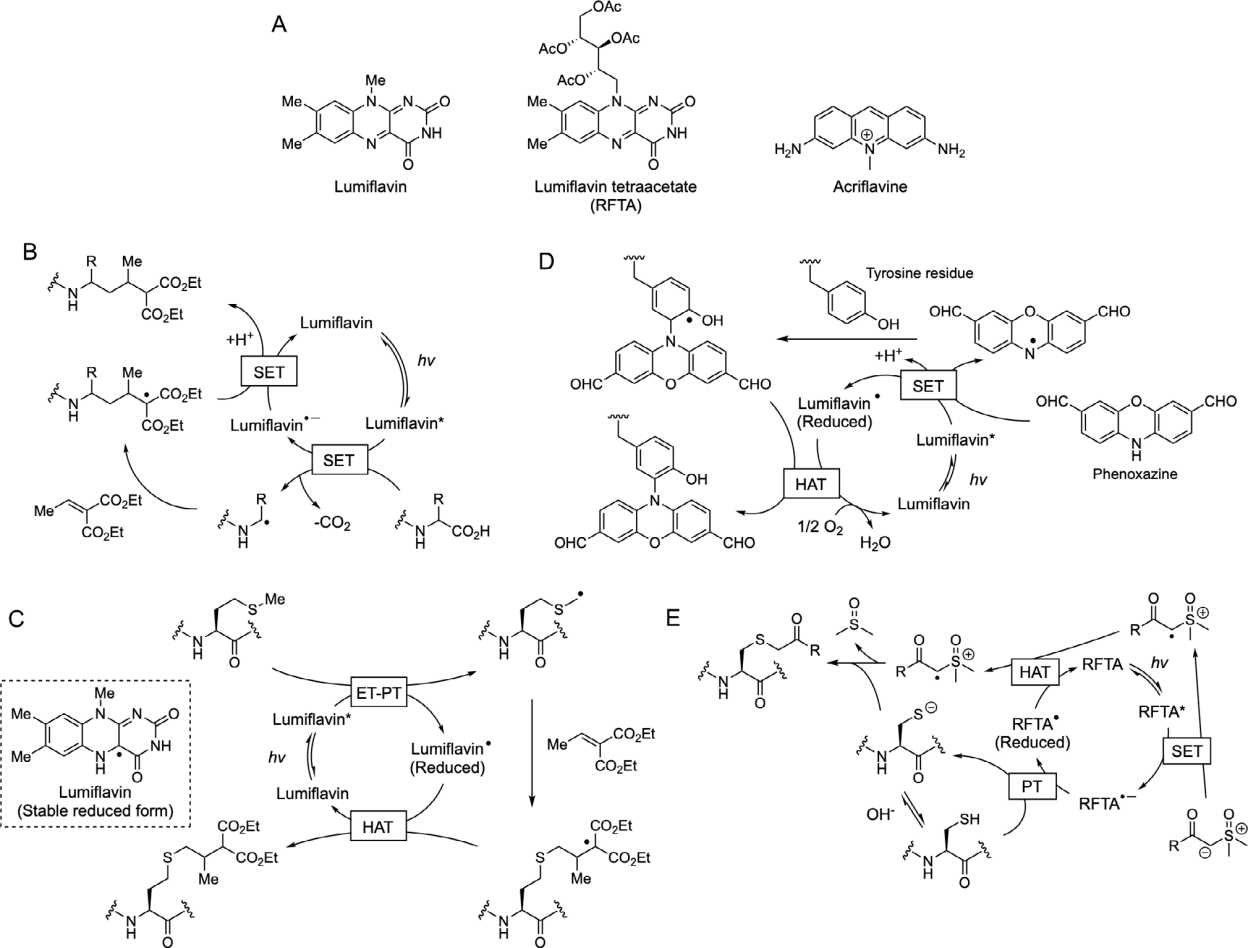
Peptide and protein modification via the SET mechanism using flavin derivatives. (A) Structures of flavin derivatives. (B) C‐Terminal modification with diethyl ethylidenemalonate. (C) Cysteine modification with diethyl ethylidenemalonate. (D) Tyrosine modification by lumiflavin‐phenoxazine system. (E) Cysteine desulfurization with sulfoxonium ylide reagents.

In contrast to metal‐based systems, flavin derivatives introduce distinct redox properties and excited‐state dynamics that enable complementary residue selectivity under physiological conditions. Flavins, exemplified by riboflavin (vitamin B_2_), are widely recognized as biologically relevant coenzymes and possess high water solubility and excellent biocompatibility [24]. These properties have recently attracted attention for the application of flavins as metal‐free photocatalysts for chemical modification of peptides and proteins. Notably, flavin catalysts can achieve reactions that are challenging with metal‐based catalysts and demonstrate high site selectivity under physiological conditions (Figure 4A).

Bloom and colleagues reported that replacing conventional photocatalysts with flavins enabled intermolecular reactions between C‐terminal carboxylic acids of peptides or proteins and labeling reagents. In these reactions, riboflavin or lumiflavin served as highly effective photocatalysts, whereas Ir or Ru complexes or other water‐soluble organic dyes showed insufficient reactivity. Mechanistically, a single‐electron oxidation of the C‐terminal carboxylate by the excited flavin catalyst, followed by decarboxylation, generated an α‐amino radical, which underwent conjugate addition to a Michael acceptor and subsequent reduction to yield the product. This reaction proceeded efficiently under physiological pH and was generally tolerant of most natural amino acids, including aspartic acid (Asp) and glutamic acid (Glu). However, the presence of Tyr, Lys, or His reduced yields due to side reactions. Such issues were mitigated by employing a strained Michael acceptor, 3‐methylene‐2‐norbornanone, enabling efficient labeling (Figure 4B) [25].

Moreover, the same group reported the first visible‐light‐mediated, Met‐selective bioconjugation. In this approach, the triplet excited state of lumiflavin induced SET at the thioether moiety of Met, generating a radical cation. Subsequent α‐deprotonation formed an α‐thiyl radical, which underwent Michael addition and hydrogen atom transfer (HAT) to afford alkylated Met. This method was applicable to diverse proteins under aqueous conditions and exhibited high selectivity over other amino acid residues (Figure 4C) [26].

Flavin catalysis has also been applied to Tyr‐selective modifications. Song and colleagues also discovered that in the presence of flavin catalysts, phenoxazines could selectively couple with Tyr residues. While electrochemical methods using related phenothiazine derivatives had been reported, these faced solubility challenges [27].

We developed an intracellular photocatalytic‐proximity labeling (iPPL) method using acriflavin as photocatalyst to analyze PPIs with high spatial resolution. In this system, acriflavin photocatalyst bound to HaloTag‐fused proteins was excited by visible light and transferred electrons via SET to nearby 1‐methyl‐4‐arylurazole (MAUra), thereby generating short‐lived radical species that engaged in proximity‐dependent coupling. These radicals reacted with proximal protein residues to form covalent bonds. Because radical generation and trapping were confined extremely locally, labeling was restricted to within a few nanometers of the photocatalyst, enabling higher spatial resolution PPI mapping compared to traditional BioID or APEX methods [28].

Hope and colleagues performed mechanistic studies on Tyr residue proximity labeling using flavin‐dependent photoredox catalysts. Upon visible‐light excitation, the flavin catalyst (riboflavin tetraacetate) reached a triplet excited state and oxidized nearby Tyr residues or phenolic probes to generate phenoxyl radicals. Radical–radical recombination subsequently formed stable covalent bonds. This pathway is thermodynamically more favorable than conventional radical addition mechanisms, and the short lifetime and localized generation of radicals enabled highly efficient and selective labeling. In the presence of oxygen, the catalyst was reoxidized, allowing continuous catalytic turnover (Figure 4D) [29].

Flavin catalysis has also been applied to Cys desulfurization reactions. Wan and colleagues reported a novel method in which sulfoxonium ylide labeling reagents were activated via SET and HAT processes. Although sulfoxonium ylides were highly stable and safe, the photoredox reaction converted them into highly reactive electrophilic species capable of modifying Cys residues with over 90% selectivity. Photophysical and mechanistic analyses revealed that flavin derivatives exhibited superior reactivity compared to Ru or Ir complexes and other photocatalysts such as Rose Bengal, generating active species via a multistep mechanism combining singlet and triplet excitation, SET, proton transfer, and HAT (Figure 4E) [30].

#### 2.1.5 Porphyrin Scaffold‐Based Photocatalysts

**FIGURE 5:**
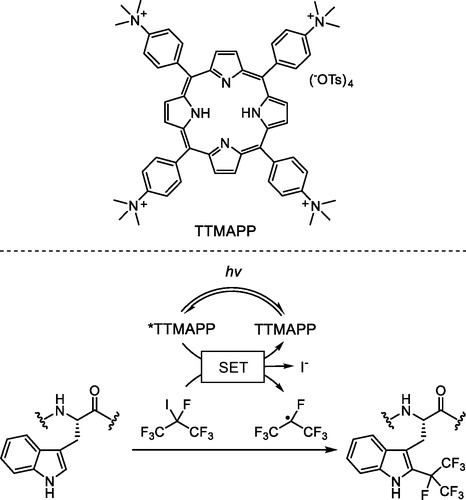
Structure of porphyrin scaffold‐based photocatalyst and tryptophan modification with iodo‐perfluoroalkyl compound.

Ryu and colleagues have reported strategies for protein labeling via the installation of fluoroalkyl groups using NIR light–mediated photoredox catalysis. Photocatalysts employed in these studies include NIR‐activatable systems such as 5,10,15,20‐tetrakis(4‐trimethylammoniophenyl)porphyrin tetra(*p*‐toluenesulfonate) (TTMAPP) and *N*′‐di‐*n*‐propyl‐1,13‐dimethoxyquinacridinium tetrafluoroborate (^n^‐Pr‐DMQA^+^). Upon NIR light irradiation, these photocatalysts were excited to their active states and could engage in SET with fluoroalkyl precursors, such as iodo‐perfluoroalkyl compounds, generating highly reactive fluoroalkyl radicals. The resulting fluoroalkyl radicals preferentially added to electron‐rich aromatic residues, particularly Trp, installing fluoroalkyl groups covalently onto protein side chains via C—C bond formation. This radical generation and subsequent addition were driven by the photoredox catalyst through the SET pathway (Figure 5). Importantly, NIR light offered deep tissue penetration, allowing these reactions to proceed efficiently even within complex cellular or tumor environments. Furthermore, the introduction of biotinylated reagents enabled the subsequent conversion of fluoroalkylated sites into detectable labels for proteomic analysis or imaging applications [31].

#### 2.1.6 Other Organic Photocatalysts

In addition to metal complexes, flavin derivatives, and porphyrin derivatives, organic photocatalysts with high structural tunability have recently emerged as important tools for peptide and protein modification. These organic photocatalysts offer not only high biocompatibility due to their metal‐free nature but also the ability to finely tune their redox and EnT properties through molecular design.

1,2,3,5‐tetrakis(carbazol‐9‐yl)‐4,6‐dicyanobenzene (4CzIPN) was a representative donor–acceptor‐type organic photocatalyst that has attracted significant attention in recent photoredox studies. The molecule consisted of a dicyanobenzene core as an electron acceptor, functionalized with four electron‐donating carbazole units, enabling the formation of long‐lived excited states under visible‐light irradiation [32]. This property arose from the effective utilization of triplet excited states via thermally activated delayed fluorescence (TADF), which in turn facilitated highly efficient SET reactions [33]. Additionally, the large redox potential of the excited state allowed for strong oxidative and reductive capability, enabling diverse photoredox transformations without the need for metal catalysts.

**FIGURE 6:**
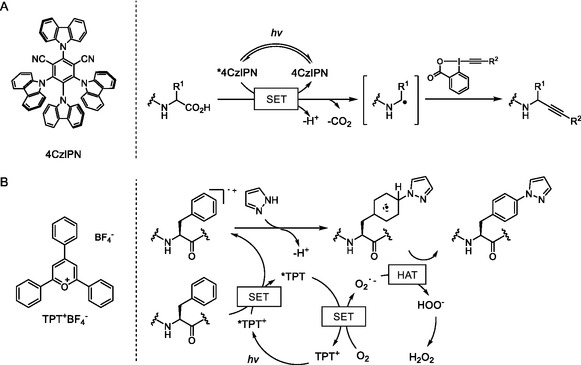
Peptide and protein modification via the SET mechanism using 4CzIPN and QCAT. (A) Structure of 4CzIPN and C‐terminal modification with hypervalent iodine alkynyl reagents (EBX reagents). (B) Structure of TPT^+^BF_4_^−^ and phenylalanine modification with pyrazole.

Garreau and colleagues reported the selective decarboxylative alkynylation of peptide C‐terminal carboxylic acids using 4CzIPN under visible‐light irradiation. In this process, photoexcited 4CzIPN selectively oxidized the C‐terminal carboxylate to generate a carboxyl radical, which rapidly released CO_2_ to form an α‐aminoalkyl radical. This radical then added to hypervalent iodine alkynyl reagents (EBX reagents), resulting in C(sp^3^)—C(sp) bond formation. Since C‐terminal carboxyl groups were more readily oxidized than the side chains of Asp or Glu, high C‐terminal selectivity was achieved (Figure 6A) [34]. This metal‐free, mild protocol represented an important late‐stage peptide modification strategy, and the same group further demonstrated that the generated α‐aminoalkyl radical could serve as a versatile intermediate for introducing diverse functional groups [35].

Moreover, Liu and colleagues designed a carbazolyl‐dicyanobenzene‐based photocatalyst (CzPN) covalently conjugated to peptides, creating a platform capable of differentiating SET and EnT processes within a single molecule. Upon visible‐light excitation, the CzPN–peptide conjugate exhibited strong redox properties, enabling radical generation at C‐terminal and cysteine residues for alkynylation or thiol–ene addition reactions. Simultaneously, high‐energy EnT processes efficiently induced nitrene formation from aryl azides, allowing for site‐selective and efficient protein modifications [36].

Triphenylpyrilium (TPT^+^) is an aromatic cationic organic photocatalyst in which three phenyl groups are attached to a pyrilium core, exhibiting extremely high oxidative potential under visible‐light excitation [37]. The excited state of TPT^+^ can oxidize substrates via SET to form radical cations, which then undergo nucleophilic addition or decarboxylation to yield the desired products. Its metal‐free nature and ability to regenerate under aerobic conditions make TPT^+^ suitable for site‐selective modification of biomolecules. Weng and colleagues demonstrated that even the typically inert phenylalanine (Phe) residues in peptides and proteins could be modified through a photoredox‐catalyzed SET process. The excited TPT^+^ photocatalyst oxidized Phe residues via SET, generating aromatic radical cations that activated the para position for nucleophilic addition of reagents such as pyrazoles (Figure 6B). This approach overcame the intrinsically low reactivity of Phe and was broadly applicable from isolated peptides to full proteins [38].

Furthermore, Choi and colleagues reported Cys‐selective modifications using quinone‐based organic photocatalysts, including practical systems such as QCAT and PEGylated QPEG. Visible‐light‐excited quinone catalysts generated thiyl radicals (RS·) from cysteine thiols via SET and/or HAT. These radicals added to electron‐deficient alkenes to form C—S bonds, and subsequent HAT stabilized the products. PEGylation of QPEG significantly enhanced solubility and biocompatibility, enabling metal‐free, visible‐light‐mediated, highly selective cysteine modifications while preserving other amino acid residues [39].

### 2.2 Photocatalysts for Energy Transfer Mechanisms

Protein modification reactions based on the EnT mechanism are characterized by the predominance of excitation EnT rather than direct electron transfer between the photocatalyst and the substrate. Instead, the process is initiated by the transfer of excitation energy from the photoexcited catalyst to the substrate or to molecular oxygen. In EnT mechanisms, the photocatalyst does not primarily function as a redox reagent; rather, it acts as a photosensitizer that efficiently generates and transfers its excited state. Consequently, reactions can be designed independently of the substrate's redox potential, which fundamentally distinguishes EnT‐based processes from those driven by SET mechanisms.

During the EnT process, energy is transferred from the triplet excited state of the photocatalyst to acceptor molecules, following two distinct pathways depending on the nature of the energy acceptor. First, EnT to molecular oxygen generates reactive oxygen species (ROS), including ^1^O_2_, which serve as highly reactive intermediates that drive protein modification reactions [40]. ^1^O_2_ exhibits strong electrophilicity while having a short lifetime and limited diffusion distance, enabling selective reactions with specific amino acid residues such as Tyr, Trp, and His, and thus allowing for spatially controlled modification [41].

Second, and of particular recent interest, photocatalysts can directly activate labeling reagent precursors through EnT mechanisms, most notably via Dexter energy transfer (DET). In this pathway, the triplet excited state of the photocatalyst transfers energy to stable precursors such as diazirines or aryl azides, inducing nitrogen extrusion to generate highly reactive carbene or nitrene intermediates. Because DET operates through a triplet–triplet exchange mechanism requiring orbital overlap, it occurs only at extremely short distances (typically <1 nm), fundamentally limiting the diffusion radius of the resulting reactive species. This distance constraint has been exploited in μMap technology, which achieves unprecedented spatial resolution (<4 nm) for proximity labeling and has emerged as a transformative approach for mapping protein microenvironments at the cell surface and in complex biological settings. The ability to generate short‐lived carbenes or nitrenes directly at the site of the photocatalyst, without relying on diffusible ROS, represents a conceptually distinct mode of EnT‐based proximity labeling with unique advantages in spatial precision.

Reaction design based on EnT mechanisms does not require strong redox potentials, which can help suppress issues associated with SET‐based approaches, such as overoxidation or non‐selective radical chain reactions. However, in ROS‐mediated processes, precise control over the amount, lifetime, and diffusion range of ROS is essential, necessitating careful optimization of both catalyst design and irradiation conditions to maintain biocompatibility.

From a spatial perspective, EnT‐based strategies offer a spectrum of labeling radii, ranging from sub‐nanometer resolution for carbene or nitrene intermediates to several nanometers for ^1^O_2_‐mediated oxidation, allowing tunable proximity mapping depending on the chosen photocatalyst–probe design. This section focuses on protein modification reactions driven by EnT mechanisms, highlighting the mechanistic differences from SET processes and discussing how EnT‐type photocatalytic reactions can offer enhanced selectivity, controllability, and potential applications in chemical biology.

#### 2.2.1 Ruthenium Complexes

**FIGURE 7:**
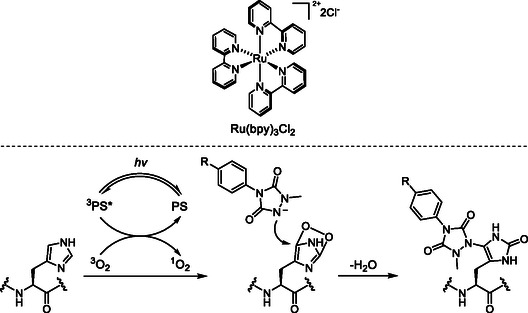
Peptide and protein modification via the EnT mechanism using Ru complex. Structure of Ru complex and histidine modification with MAUra. PS: photosensitizer.

Ru(bpy)_3_ complex is one of the earliest photocatalysts used for peptide and protein modification. In contrast to SET‐type roles, Ru(bpy)_3_ complexes such as those used by Lee and colleagues efficiently sensitize the generation of ^1^O_2_ under visible light, acting as potent photosensitizers that produce ROS capable of inactivating proteins and facilitating oxidative modification pathways [42]. We reported a His‐selective proximity labeling method utilizing ^1^O_2_. Upon visible‐light excitation, the photosensitizer transferred energy to triplet oxygen (^3^O_2_) via triplet–triplet energy transfer (TTET), generating ^1^O_2_. The produced ^1^O_2_ reacted with the imidazole ring of histidine, inverting its electronic polarity, allowing external nucleophilic labeling reagents to selectively add to the site (Figure 7). Due to the short lifetime and limited diffusion distance of ^1^O_2_, only histidine residues in close proximity to the photosensitizer were labeled, enabling site‐selective, mild, and highly biocompatible protein modification [43].

#### 2.2.2 Iridium Complexes

**FIGURE 8:**
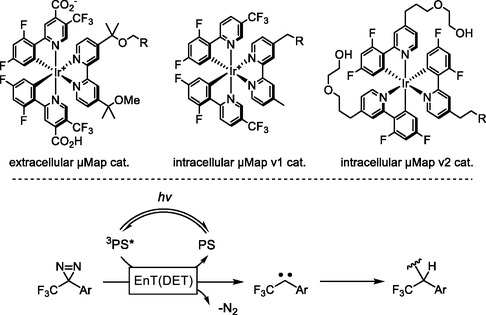
Structures of Ir complexes and any amino acids modification with aryl diazirine derivatives.

Ir complexes are widely utilized as photoredox catalysts and, compared to organic dyes, exhibit long‐lived excited states, rendering them highly sensitive to EnT reaction (Figure 8). Depending on the reaction conditions, including solvent, light source, and pH, EnT can occur in combination with TTET reaction.

A representative example of Ir‐catalyzed photo‐labeling is the use of diazirine derivatives. Diazirine is photoactivatable labeling reagent, typically activated by UV light for peptide or protein modification; however, it can also be activated under visible light via Ir‐catalyzed TTET. Geri and colleagues reported that an Ir[dF(CF_3_)ppy]_2_(dtbbpy)(PF_6_) catalyst activated a diazirine probe to generate a short‐lived carbene intermediate via a DET mechanism. This carbene had an extremely short half‐life and reacted only within 4 nm of its target, enabling both high site‐selectivity and biocompatibility (Figure 8). Because DET did not involve electron transfer or the generation of ROS, it minimally perturbed the cellular environment, efficiently labeling only molecules in close proximity to the photocatalyst [44, 45, 46].

#### 2.2.3 Osmium Complexes

**FIGURE 9:**
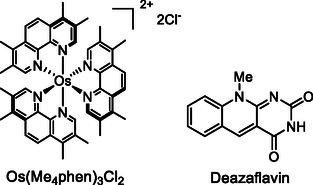
Structure of Os complex and deazaflavin.

To enhance biocompatibility while improving light penetration into deep tissues, the development of metal‐based photocatalysts suitable for amino acid residue modification under red‐ and NIR‐light irradiation has been actively pursued. In particular, osmium (Os) complexes, which possess long‐lived excited states and high triplet energies, have proven effective for proximity‐dependent protein labeling in the visible‐to‐red light region (Figure 9).

Shah and Rovis reported a high‐resolution protein labeling strategy using an Os(II) complex photocatalyst under red‐light excitation, in which aryl trifluoromethyl diazo compounds were activated via an EnT mechanism. The key to this reaction was the EnT from the excited triplet state of the Os(II) complex to the diazo compound, with DET rather than SET dominating the process. Indeed, the reduction potential of the diazo compounds was highly negative, and the reaction efficiency was determined by triplet energy matching rather than the reducing power of the catalyst, thereby excluding an electron‐transfer pathway. Upon receiving energy, the diazo compound was excited and rapidly extruded nitrogen to generate a carbene intermediate. This short‐lived carbene inserted into nearby amino acid side chains (O–H, N–H, S–H bonds), enabling protein labeling. Furthermore, the electronic properties of the diazo compound influenced the spin state of the generated carbene. Electron‐donating substrates predominantly yielded highly reactive singlet carbenes, whereas electron‐withdrawing substrates increased the proportion of triplet carbenes, which could lead to a higher incidence of side reactions [47].

#### 2.2.4 Flavin Derivatives

Although many flavin derivatives have traditionally been employed for peptide and protein modification via SET mechanism, recent efforts have focused on developing modification strategies based on EnT mechanism (Figure 9).

Crocker and colleagues reported the use of deazaflavin as a photocatalyst for EnT‐based proximity labeling in live cells. Upon light excitation, deazaflavin underwent intersystem crossing from its singlet excited state (S_1_) to the triplet excited state (T_1_) and subsequently activated nearby labeling reagents via DET. Activated labeling reagents, such as phenylazide derivatives, could covalently attach to protein residues, including Cys, Lys, Tyr, or to nucleobases in RNA. Notably, this process did not proceed through SET and did not generate electron transfer or ROS, resulting in high biocompatibility. Furthermore, the short‐lived excited state ensured that only molecules in close proximity to the photocatalyst were labeled, providing high spatial resolution and site selectivity [48].

#### 2.2.5 Xanthene Scaffold‐Based Photocatalysts

**FIGURE 10:**
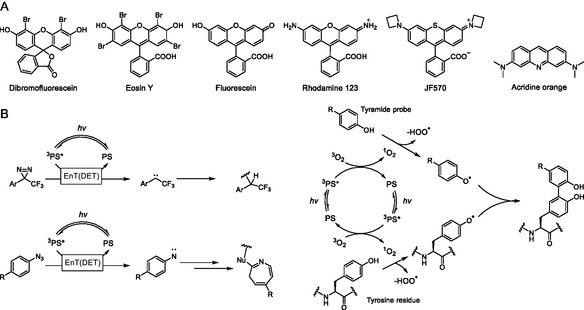
Peptide and protein modification via the EnT mechanism using xanthene scaffold‐based photocatalysts. (A) Structure of xanthene scaffold‐based photocatalysts. (B) Any amino acids and tyrosine modifications with aryl diazirine, aryl azido, and aryl phenol derivatives, respectively.

Organic dyes containing the xanthene scaffold, such as fluorescein, have been extensively studied over the years (Figure 10A). However, the properties of their excited states remain incompletely understood due to complex equilibria influenced by solvent and pH. Generally, halogenated fluorescein derivatives, including Eosin Y, dibromofluorescein, and rose bengal, exhibit rapid intersystem crossing (ISC) with high efficiency due to the heavy‐atom effect, favoring EnT pathways that generate ^1^O_2_ (Figure 10B).

Wang and colleagues reported a photooxidation‐driven proximity labeling strategy using dibromofluorescein with a triphenylphosphine moiety (TPP‐AcDBF). Upon visible light irradiation, the dibromofluorescein was excited and generated ^1^O_2_ from ground‐state ^3^O_2_. The highly electrophilic and short‐lived ^1^O_2_ selectively oxidized His residues within mitochondria. The resulting oxidized intermediates then reacted with nucleophilic labeling reagents, such as propargylamine, to covalently label the target protein. This ^1^O_2_‐dependent oxidation‐induced labeling proceeded as a localized reaction restricted to the vicinity of the photocatalyst, achieving high spatiotemporal selectivity and biocompatibility [49].

Lin and colleagues reported the MultiMap method using Eosin Y‐conjugated antibodies for proximity protein labeling. Upon photoexcitation, Eosin Y activated labeling reagents (diazirines, aryl‐azides, or phenols) via an EnT mechanism, generating highly reactive intermediates such as carbenes, nitrenes, or phenoxy radicals with distinct lifetimes. This allowed proximity labeling over a range of distances depending on the intrinsic reactivity of the probe [50].

Wang and colleagues demonstrated that xanthene‐based dyes, including acridine orange, fluorescein, and Rhodamine 123, could function as photocatalysts under visible light. Notably, Rhodamine 123 accumulated in mitochondria and, upon visible‐light excitation, activated arylazides to generate nitrene intermediate. This intermediate was further converted into highly reactive species, such as benzazirines or ketenimines, which covalently reacted with nucleophilic amino acid residues in proteins. Since the activation primarily proceeded via EnT, the reaction occurred locally and in close proximity, enabling high spatiotemporal resolution labeling of mitochondrial proteins [51].

Becker and colleagues developed the photosensitizer‐dependent oxidation and capture by amine (POCA) platform using JF_570_ as a photocatalyst for intracellular proximity labeling. JF_570_ was introduced either as a HaloTag ligand conjugate (JF_570_‐HTL) or a cholesterol‐binding probe (chol‐JF_570_). Upon visible‐light excitation, JF_570_ generated ^1^O_2_ via EnT. The ^1^O_2_ oxidized nearby chemical species to form reactive intermediates, which were subsequently captured by amine reagents such as propargylamine or 3‐ethynylaniline, allowing covalent labeling of proteins or lipid‐associated molecules in close proximity. The labeling proceeded locally and rapidly, enabling high spatial resolution mapping of cholesterol‐binding proteins and their associated complexes [52].

#### 2.2.6 Porphyrin and Chlorin Scaffold‐Based Photocatalysts

**FIGURE 11:**
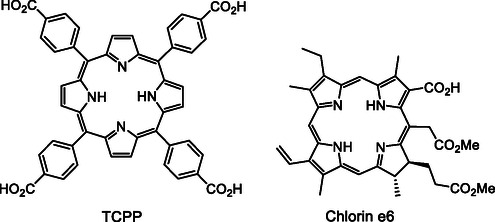
Structures of porphyrin‐ and chlorin‐scaffold‐based photocatalysts.

Porphyrins and chlorins are ubiquitous chromophores that efficiently absorb long‐wavelength light (>600 nm). To enhance biocompatibility and improve tissue penetration, extensive efforts have been devoted to developing photosensitizers based on these scaffolds (Figure 11).

Qianqian and colleagues applied photocatalytic proximity labeling using a 2D nanophotocatalyst platform for surface proteome analysis in living cells. The underlying photocatalyst was tetra(carboxyphenyl)porphyrin (TCPP) immobilized on a 2D nanomaterial, which stably anchored to the cell surface to create a localized photoreactive environment. Upon visible‐light irradiation, excited TCPP generated ^1^O_2_ via EnT from its excited state to the ground state. The highly reactive ^1^O_2_ oxidized proximal protein residues, such as His, forming reactive intermediates. These intermediates subsequently reacted covalently with capture handles, such as amine or biotinylated reagents, resulting in tagging of the target proteins. This mechanism localized the reaction to the vicinity of the photocatalyst, enabling high spatiotemporal resolution mapping of the cell‐surface interactome even within complex cellular environments [53].

Lou and colleagues reported a biocompatible deep‐red light‐driven photocatalytic proximity labeling method, CAT‐Tissue. In this system, the photocatalyst was chlorin e6 conjugated to a nanobody. Upon excitation with deep‐red light (650–700 nm), chlorin e6 efficiently generated ^1^O_2_ via EnT. The use of deep‐red light enhanced tissue penetration and minimized off‐target reactions, allowing precise in situ labeling under physiological conditions. The generated ^1^O_2_ oxidized nearby protein residues or other biomolecular side chains to form highly reactive intermediates, which reacted with labeling reagents such as biotin–aniline to covalently tag target molecules. The chlorin e6‐driven reaction primarily proceeded through ^1^O_2_ generation via EnT, enabling localized and rapid labeling under light irradiation. This approach allowed high‐resolution visualization of in situ tumor–immune cell proximity interactions in both spatial and temporal dimensions [54].

#### 2.2.7 Other Organic Photocatalysts

Although the detailed reaction mechanisms are not yet fully elucidated, various organic photosensitizers capable of modifying peptides and proteins via EnT have been reported. In this section, we focus on such organic photocatalysts and highlight representative applications.

**FIGURE 12:**
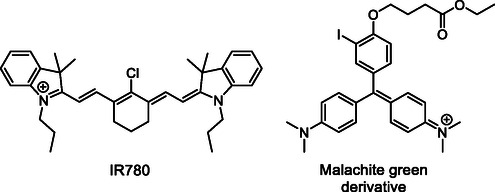
Structures of IR780 and malachite green.

Zeng and colleagues developed a proximity labeling method for the mitochondrial proteome in living cells using NIR light. The photosensitizer was employed, IR780, a heptamethine cyanine dye with NIR absorption, which selectively accumulated in mitochondria (Figure 12). Upon NIR light irradiation (700–800 nm), excited IR780 transferred energy to O_2_ via EnT to generate ^1^O_2_. This highly oxidizing species reacted with proximal protein residues, such as His, forming reactive intermediates. These intermediates rapidly reacted with nucleophilic labeling reagents, such as alkynyl‐amines (e.g., propargylamine), introducing covalent alkyne tags onto the proteins. Subsequent copper‐catalyzed azide–alkyne cycloaddition (CuAAC) enabled the installation of biotin handles for downstream analysis or purification [55].

Furthermore, Ren and colleagues reported a gene‐encoded NIR photocatalyst‐based proximity labeling method, termed FLAPP. In this system, the target protein was fused to the fluorogen‐activating protein dL5, and the photosensitizer was covalently linked to iodinated malachite green (Figure 12). Upon NIR light irradiation, the complex was excited and generated ^1^O_2_ via EnT from O_2_. The generated ^1^O_2_ oxidized proximal residues, such as His, to form highly reactive intermediates, which subsequently reacted with externally supplied nucleophilic labeling reagents, such as biotin‐aniline or propargylamine. This approach enabled highly localized and selective protein labeling in living cells [56].

#### 2.2.8 Genetically Encoded Protein‐Based Photocatalysts

In addition to organic photocatalysts, various protein‐based photosensitive molecules have been engineered and utilized as biological tools. In particular, fluorescent proteins can be endowed with photocatalytic functionality through biological modifications, making them applicable for peptide and protein modification. A representative example is mini singlet oxygen generator (miniSOG). miniSOG is a small photosensitizing protein derived from flavoproteins, comprising approximately 106 amino acids with a molecular weight of ∼12 kDa [57]. While retaining properties similar to conventional fluorescent proteins, miniSOG generates ^1^O_2_ upon blue‐light irradiation (∼450 nm) [58]. Although it was primarily used for contrast generation in optical and electron microscopy, as well as for light‐induced cell ablation and phototoxicity experiments, its small size and ease of intracellular delivery make it a versatile tool for simultaneous fluorescence imaging and oxidative modification.

Zhai and colleagues fused miniSOG to proteins of interest(POIs) and reported a proximity labeling method termed photoactivation‐dependent proximity labeling (PDP). Upon blue‐light irradiation (∼460 nm), the excited miniSOG transferred energy to O_2_ via an EnT process, generating ^1^O_2_. The resulting ^1^O_2_ oxidized nucleophilic residues such as His in nearby proteins, forming highly reactive intermediates. These intermediates reacted covalently with amine‐containing chemical probes (e.g., aniline derivatives, propargylamine). The probes carried alkyne handles, allowing subsequent introduction of detection tags such as biotin or fluorophores via click chemistry, facilitating mass spectrometry or imaging applications. The EnT‐driven ^1^O_2_ generation and oxidative capture reaction cascade enabled precise temporal and spatial control of protein network profiling [59].

More recently, Sun and colleagues reported bioluminescence resonance energy transfer (BRET)‐ID, a light‐independent photocatalytic activation technique. In BRET‐ID, a genetically fused NanoLuc and a photosensitizing domain (e.g., SOPP3) formed a photocatalytic complex. Upon addition of the substrate furimazine, bioluminescence was generated by NanoLuc, and the emitted energy was transferred to the photosensitizing domain via BRET. The excited photosensitizing domain then produced ^1^O_2_ from O_2_ via EnT, oxidizing nearby protein residues (mainly His) to form reactive intermediates. These intermediates reacted with alkynyl probes for covalent protein labeling, and subsequent click chemistry enabled the introduction of biotin or other detection tags, allowing downstream applications in mass spectrometry and imaging [60].

### 2.3 Determinants of Mechanistic Pathway Selection in Photocatalytic Protein Modification

As discussed in Sections 2.1 and 2.2, photocatalytic protein modification reactions are broadly classified into SET and EnT mechanisms. However, increasing evidence indicates that these pathways are not mutually exclusive; rather, they can operate competitively or in parallel depending on the reaction environment. Notably, in Ru‐ and Ir‐based systems, both SET‐mediated radical formation and EnT‐driven processes such as ^1^O_2_ generation can occur from the same photoexcited state. For example, Ru‐based photocatalysts have been shown to mediate tyrosine modification via SET while simultaneously enabling histidine oxidation via ^1^O_2_, with the dominant pathway governed by kinetic and environmental factors. These observations highlight the importance of establishing a unified framework for predicting mechanistic pathway selection.

#### 2.3.1 Photocatalyst Properties: Redox Potential and Triplet Energy

The intrinsic photophysical properties of the photocatalyst constitute the primary determinant of the operative mechanism. SET pathways require that the excited‐state redox potential of the photocatalyst be sufficient to oxidize or reduce the substrate or labeling reagent. In contrast, EnT pathways depend on the triplet energy and lifetime of the excited state, which must be compatible with energy transfer to molecular oxygen or to suitable acceptor molecules. Photocatalysts such as Ru(bpy)_3_ and Ir complexes possess both strong redox potentials and accessible triplet excited states, enabling dual SET/EnT reactivity. In such systems, the dominant pathway is dictated by the relative kinetics of electron transfer versus energy transfer. Conversely, photocatalysts with weaker redox potentials but efficient intersystem crossing, such as xanthene‐based dyes, generally favor EnT‐dominated processes.

#### 2.3.2 Oxygen Dependence

Oxygen availability plays a decisive role in pathway selection. EnT processes that proceed via ^1^O_2_ generation are inherently oxygen‐dependent, relying on triplet–triplet energy transfer from the photocatalyst to ground‐state oxygen (^3^O_2_). Under aerobic conditions, this pathway is often favored, leading to selective oxidation of residues such as His, Trp, and Tyr. In contrast, SET pathways can proceed in both the presence and absence of oxygen, although oxygen may act as a quencher of excited states or participate in secondary oxidation reactions. Therefore, modulation of oxygen concentration provides a practical strategy to bias the reaction toward EnT (oxygen‐rich conditions) or SET (oxygen‐limited conditions).

#### 2.3.3 Substrate Proximity and Spatial Constraints

Spatial organization is a critical determinant, particularly in protein modification and proximity labeling applications. SET processes typically involve diffusible radical intermediates whose effective range depends on their lifetime and the local chemical environment. In contrast, EnT processes—especially those involving DET—require direct orbital overlap between the photocatalyst and the energy acceptor, imposing a fundamental distance constraint on the activation step itself. The resulting reactive intermediates (carbenes, nitrenes) are themselves short‐lived, further confining the spatial extent of modification. Quantitative measurements of labeling radii achieved by each system in biological contexts are discussed in Section 3.7. Accordingly, the relative positioning of the photocatalyst and target residues strongly influences mechanistic outcomes. When electron‐rich residues such as Tyr or Trp are located in close proximity to the photocatalyst, rapid SET can dominate due to favorable electron transfer kinetics. Conversely, when energy transfer to oxygen or to a bound labeling reagent is more efficient, localized EnT pathways, including ^1^O_2_ generation or carbene/nitrene formation, become predominant. This spatial dependence underlies the high resolution achievable in proximity labeling strategies.

#### 2.3.4 Light Intensity and Wavelength

Irradiation conditions further modulate the balance between SET and EnT pathways. Light intensity influences the steady‐state population of excited photocatalysts, thereby affecting the rates of both processes. While higher light intensity can enhance overall reactivity, it may also increase undesired side reactions, such as overoxidation in SET‐based systems. The wavelength of irradiation determines the excited‐state energetics of the photocatalyst. Shorter wavelengths (e.g., blue light) generally provide higher‐energy excited states with stronger redox capabilities, thereby favoring SET processes. In contrast, longer wavelengths (red to near‐infrared) are associated with lower excitation energy but improved tissue penetration and reduced phototoxicity, and are often employed in EnT‐dominated systems, particularly those involving ^1^O_2_ generation or triplet sensitization of labeling reagents.

### 2.4 Mechanistic Comparison of Photocatalytic Proximity Labeling Platforms

In Section 2, recent advances in photocatalyst‐mediated peptide and protein modification were summarized, highlighting the transition from conventional UV‐driven strategies to visible‐ and NIR‐light‐enabled photocatalysis. Photocatalytic modification can be broadly categorized into SET and EnT mechanisms, which differ fundamentally in the nature of their reactive intermediates and spatial behavior. SET‐based approaches generate radical species through redox processes, enabling efficient and versatile modification of amino acid residues such as Tyr, Trp, His, His, and C‐terminal. In contrast, EnT‐based approaches generate short‐lived carbene or nitrene intermediates from various labeling reagents via DET, or the direct production of ROS (e.g.,^1^O_2_) by a photosensitizer. These mechanistic differences directly influence labeling selectivity, reaction scope, and spatial confinement. Importantly, because proximity labeling is inherently a spatially defined process, quantitative parameters such as intermediate lifetime, diffusion distance, and effective labeling radius are critical for evaluating and comparing different systems. The reactive intermediates summarized in Table 1 differ markedly in their intrinsic lifetimes, which range from picoseconds for carbenes to microseconds for ^1^O_2_. Because shorter‐lived intermediates react closer to their site of generation, intermediate lifetime imposes an upper bound on the spatial extent of labeling. However, translating these chemical lifetimes into effective labeling radii requires consideration of additional biological variables—including catalyst localization strategy, tether dimensions, molecular crowding, and redox buffering—that modulate diffusion in cellular environments. Experimentally measured labeling radii under biological conditions are therefore discussed in Section 3.7 and summarized in Table 2. The intrinsic chemical properties of these reactive intermediates—including lifetimes and diffusion distances—are summarized in Table 1. However, the effective biological resolution is not solely determined by these chemical diffusion limits but is also influenced by catalyst localization strategy, the dimensions of the molecular tether, protein dynamics, and the cellular environment. These biological performance considerations, together with a direct comparison to enzyme‐based methods, are discussed in Section 3.7 and Table 2.

**TABLE 1:** Reactive intermediates in photocatalytic protein modification.

Reactive intermediate	Mechanism	Typical lifetime	Target residues	Key features
Radical (Photocatalytic activation)	SET/HAT	ns–μs	Tyr, Trp, His, Met, Cys, C‐terminal	Versatile, diverse labeling
Singlet oxygene (^1^O_2_)	EnT	μs	His, Tyr, Trp	Diffusible, oxidative labeling
Carbene	EnT (DET)	ps–ns	Broad (C—H, N—H, O—H insertion)	Ultra‐local labeling
Nitrene	EnT	ns–μs	Broad residues	Highly electrophilic, less selective labeling
Aminyl radical	SET	ns	Nearby residues	Extremely short‐range labeling

**TABLE 2:** Quantitative comparison of enzyme‐based and photocatalytic proximity labeling methods in biological systems. Intrinsic intermediate lifetimes and diffusion distances are detailed in Table 1; the present table focuses on experimentally measured labeling radii and operational parameters.

Method	Category	Labeling radius	Temporal resolution	Reactive intermediate	Activation trigger	Catalyst localization	
Enzyme‐based methods							
BioID	Enzymatic	∼10 nm [61]	12–24 h	Biotin–AMP	Biotin (constitutive)	Genetic fusion	
TurboID	Enzymatic	∼35 nm [62]	10 min–1 h	Biotin–AMP	Biotin (constitutive)	Genetic fusion	
APEX/HRP	Enzymatic	∼200 nm [63]; FWHM 269 ± 41 nm [64]	∼1 min	Phenoxyl radical (tyramide)	H_2_O_2_	Genetic fusion/Ab conjugation	
Photocatalytic methods							
μMap (carbene)	Photocatalytic (DET)	∼4 nm intrinsic; FWHM 54 ± 12 nm (with Ab) [64]; max 11 nm (direct conjugation) [65]	Seconds–minutes	Carbene (from diazirine)	Visible light (blue, 450 nm)	Antibody/ligand [44]	
SET–nitrene (PhN_3_)	Photocatalytic (SET)	FWHM 119 ± 33 nm (PhN_3_); FWHM 68 ± 17 nm (PhF_4_N_3_) [64]	Seconds–minutes	Nitrene (from aryl azide)	Visible light (blue)	Antibody/ligand	
iPPL (Ru/MAUra)	Photocatalytic (SET)	∼6 nm (estimated from histone interactome) [28]	Seconds–minutes	Tyrosyl radical (MAUra coupling)	Visible light (blue)	Genetic fusion (HaloTag)	
DET‐based (μMap)	Photocatalytic (EnT/DET)	<1 nm (orbital overlap required)	Seconds	Direct EnT (no diffusible intermediate)	Visible light	Antibody/ligand [44]	
^1^O_2_−mediated	Photocatalytic (TTET)	Limited by ^1^O_2_ diffusion (∼100 nm in water)	5–300 s [66, 67]	Singlet oxygen (^1^O_2_)	Visible light (blue t/green/red, 460–660 nm)	Ligand/nanomaterial Genetic fusion	

Beyond mechanistic classification, cross‐cutting functional comparisons across different catalyst platforms reveal convergent strategies for residue‐selective modification. For example, tyrosine labeling has been achieved using Ru complexes, flavins, and organic photocatalysts via both SET and EnT pathways, while tryptophan and cysteine modifications have been realized through distinct but mechanistically related processes. These observations indicate that reaction outcomes are governed not only by catalyst identity but also by the interplay between reaction mechanism, substrate reactivity, and spatial constraints. Organizing these transformations by target residue and functional outcome, rather than solely by catalyst type, provides a more unified framework for comparison (Table 3).

**TABLE 3:** Comparison of amino acid‐selective modification strategies.

Target residue	Representative catalyst	Mechanism	Reactive intermediate	Key features/remarks
Tyr	Ru(bpy)_3_, Ru derivatives	SET	Tyr radical/phenoxyl radical	Classical system; high efficiency; proximity enhances selectivity
	Flavin (riboflavin, lumiflavin)	SET	Phenoxyl radical	High biocompatibility; mild conditions
	Xanthene dyes (e.g., Eosin Y)	EnT	^1^O_2_	ROS‐mediated oxidation; diffusion‐limited selectivity
	Peroxidase (APEX)	Enzymatic	Phenoxyl radicals	Tyr‐selective labeling; fast but oxidative
Trp	Ru‐TAP	SET	Trp radical cation	High selectivity due to controlled radical recombination
	Ir complexes	SET	Trp radical/β‐radical	Enables β‐functionalization; tunable via ligand design
	Porphyrin	SET	Carbon‐centered radicals	Compatible with deep tissue (NIR activation)
Cys	Ru (With trapping reagent)	SET	Thiyl radical	Selectivity influenced by proximity rather than intrinsic reactivity
	Quinone‐based catalysts (QCAT, QPEG)	SET/HAT	Thiyl radical	Metal‐free; high selectivity; good biocompatibility
	Flavin systems	SET/HAT	Sulfur‐centered radical	Enables desulfurization and diverse transformations
His	Ru(bpy)_3_	EnT	^1^O_2_	High selectivity via imidazole oxidation
	Xanthene dyes/porphyrins	EnT	^1^O_2_	Widely used in proximity labeling platforms
Lys	Biotin ligase (BioID)	Enzymatic	Biotin‐AMP	Lys‐selective labeling; slow kinetics
C‐terminus	Flavin/4CzIPN	SET	α‐amino radical	Enables late‐stage functionalization
Any (Multiple)	Ir (Diazirine activation)	EnT (DET)	Carbene	Ultra‐high spatial resolution; no ROS involvement
	Os complexes (Diazo activation)	EnT or SET/DET	Carbene	Red‐light activation; deep tissue compatibility
	Flavin (Deazaflavin)	EnT (DET)	Nitrene	Metal‐free; high spatial precision
	Sn–chlorin e6 (Aryl azido activation)	SET	Aminyl radical/nitrene‐like	Red‐light activation; intracellular compatibility
	Ru, flavin, Ir, xanthene (With labeling reagents)	SET/EnT	Radicals/^1^O_2_/carbene	Choice of mechanism defines labeling radius and selectivity
C‐terminus	Flavin/4CzIPN	SET	α‐amino radical	Enables late‐stage functionalization

Overall, integrating mechanistic insight with quantitative and biological performance considerations will be essential for the continued advancement of photocatalytic strategies in chemical biology and biomedical research.

## 3 Applications of Photocatalytic Proximity Labeling Across Biological Complexity

This chapter focuses on how these chemistries described in Chapter 2 have been implemented across biological systems of increasing complexity, emphasizing experimental outcomes rather than mechanistic principles.

Understanding how these variables scale with biological complexity is therefore essential for interpreting proximity labeling data and for selecting appropriate platforms for specific biological questions. This chapter progresses from simplified to increasingly realistic biological systems, spanning purified proteins, solid‐supported platforms, cell surfaces, intracellular environments, ex vivo tissues, and finally in vivo applications enabled by advanced light‐delivery strategies.

Across these sections, emphasis is placed not only on technical advances but also on conceptual insights: how different reactive intermediates define spatial resolution, how catalyst localization strategies dictate biological specificity, and how labeling radius should be interpreted in relation to molecular crowding and redox buffering. Together, the studies surveyed in this chapter illustrate how photocatalytic proximity labeling has evolved from a mechanistic curiosity into a versatile chemical platform capable of interrogating protein interactions across biological scales—from isolated biomolecules to living systems.

### 3.1 Application to Purified Proteins

Applications to purified proteins represent the foundational stage at which photocatalytic proximity labeling strategies were first validated and benchmarked. Under well‐defined in vitro conditions, these systems enable direct assessment of labeling selectivity, spatial confinement, and compatibility with native protein structures, providing essential reference points for later applications in complex biological environments. Representative photocatalyst‐based proximity labeling systems developed in vitro, including their photocatalysts, labeling reagents, target residues, and applications, are summarized in Table 4.

**TABLE 4:** Photocatalyst‐based proximity labeling developed in vitro systems and applications.

References	Catalyst structure	Labeling reagent	Target amino acid residue	Application
Sato and Nakamura [15], Sato et al. [68]	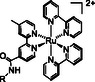	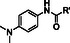	Tyr	Target‐selective labeling, closo‐dodecaborate cluster modification
Beard et al. [16]	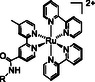	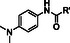	Cys	Target‐selective labeling
Sato et al. [69]	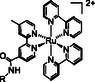	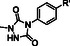	Tyr	Target‐selective labeling
Li et al. [71]	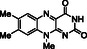	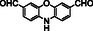	Tyr	Site‐selective modification
Bloom et al. [25]	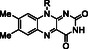	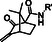	C‐term	Protein C‐term modification
Garreau et al. [34]	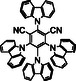	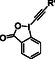	C‐term	Protein C‐term modification
Kim et al. [26]	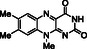	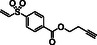	Met	Site‐selective protein modification
Nakane et al. [43]	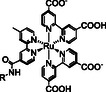	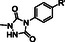	His	Fc‐selective antibody modification
Nakane et al. [19, 72]	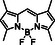	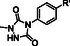	His	Fc‐selective antibody modification
Okamoto et al. [18]	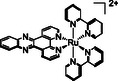	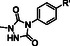	Tyr (RuDppz), His (RuDppz ⊂ RFBP)	Switching of target amino acid residues

Early studies employing Ru(bpy)_3_‐based photocatalysts demonstrated that visible‐light‐driven photoredox reactions could be harnessed for proximity‐dependent protein labeling with high selectivity [15, 16]. These results provided the first experimental validation that photocatalytic labeling efficiency decays sharply with distance from the catalyst‐binding site, allowing spatially confined modification at the nanometer scale. Building on this foundation, we attached a phenylenediamine probe that forms covalent bonds with Tyr under radical reaction conditions to a *closo*‐dodecaborate cluster and used Ru(bpy)_3_ with persulfate to label bovine serum albumin (BSA) [68].

Subsequent studies refined this concept by optimizing labeling reagents to enhance proximity dependence. Systematic screening using distance‐controlled molecular ruler substrates identified MAUra as a reagent that functions efficiently only within very short distances from the photocatalyst [69]. Mass spectrometric analysis of labeled proteins revealed that covalent modification was confined to residues located in the immediate vicinity of the ligand‐binding site, consistent with highly localized reactivity arising from the rapid quenching and short effective life time of MAUra‐derived radical intermediates [70].

Purified protein systems also enabled expansion of residue scope beyond tyrosine. Flavin‐based photocatalysts were applied to C‐terminal carboxylate modification via decarboxylative pathways, providing site‐selective installation of functional handles for conjugation and immobilization [25, 34]. Related photoredox strategies enabled methionine‐selective C—C bond formation under mild aqueous conditions while largely preserving protein structure and activity [26]. In a complementary implementation, lumiflavin‐catalyzed photoredox reactions enabled selective coupling of surface‐exposed Tyr residues to phenoxazine dialdehydes under blue light, providing aldehyde handles for subsequent installation of fluorophores, biotin, and click chemistry partners across a range of native proteins [71].

Importantly, purified protein studies revealed that residue selectivity in photocatalytic labeling can be modulated by the local microenvironment of the photocatalyst. Incorporation of photocatalysts into protein scaffolds or binding pockets enabled switching between SET‐ and EnT‐dominated pathways, thereby controlling whether tyrosine or histidine residues were preferentially modified [18]. Consistent with this principle, His‐selective MAUra labeling was successfully combined with BODIPY‐based photosensitizers tethered to ligands or HaloTag, enabling selective histidine labeling of target proteins in cell lysates and Fc‐selective modification of native antibodies under mild irradiation conditions [72].

Another photocatalytic proximity labeling approach, μMap, originally developed for live‐cell surface applications, has also been validated on purified proteins for ligand‐binding‐site identification. In this platform, a ligand‐tethered iridium photocatalyst activates diazirine probes under blue light to generate short‐lived carbenes that label residues in immediate proximity to the binding site, enabling site‐resolved contact mapping on purified proteins and simple complexes [73].

Together, these in vitro studies define a range of photocatalytic labeling handles, including Tyr, His, Met, C‐terminal carboxylates, and carbene‐reactive sites, and establish how catalyst identity, localization, and reactive intermediate lifetime determine labeling radius and selectivity (Table 4). The high degree of experimental control in purified protein systems enables detailed mass spectrometric mapping and provides a benchmark for subsequent applications in more complex biological settings.

### 3.2 Proximity Labeling on Beads

Photocatalytic labeling on beads and in cell lysates is a natural extension of in vitro chemistry to proteome‐wide analysis. In this regime, photocatalysts are immobilized on solid scaffolds such as magnetic beads or nanoparticles. After irradiation, proteins in close proximity to the scaffold are covalently tagged and identified by mass spectrometry.

Building on our established Ru(bpy)_3_‐catalyzed Tyr labeling chemistry and the highly proximity‐dependent phenylenediamine and MAUra probes described earlier, we extended this approach to solid‐phase platforms to enable proteome‐wide target identification. We prepared fine magnetic beads co‐immobilized with small‐molecule ligands such as benzenesulfonamide or lactose and Ru photocatalyst derivatives [74, 75]. Importantly, to minimize non‐specific protein adsorption observed with highly cationic Ru complex, we employed an anionic Ru complex containing 2,2′‐bipyridine‐4,4′‐dicarboxylic acid (Dcbpy), which reduces background while preserving efficient photocatalysis.

In particular, the bead‐bound RuDcbpy complex and multivalent lactose ligands were functionalized together on magnetic beads for the labeling of endogenous lectins.

In A431 cell lysate, lactose/RuDcbpy beads (5.0 mg/mL) combined with 500 μM MAUra‐desthiobiotin (DTB) at a total protein concentration of 3.0 mg/mL enable enrichment and nanoLC‐MS/MS identification of endogenous galectin‐1 and galectin‐3, together with confirmation by western blot. Competition with excess free lactose suppresses labeling and reveals that multivalent presentation on the beads increases apparent affinity by ∼10^4^‐fold compared with monomeric lactose (IC_50_ 0.398 µM vs. 3.87 mM). Despite this substantial affinity enhancement through multivalent interactions, conventional affinity chromatography still fails to capture these galectin‐lactose complexes due to the requirement for stringent washing steps that disrupt weak millimolar‐range interactions. In contrast, proximity labeling circumvents this limitation by covalently tagging proteins while they are transiently bound to the beads, eliminating the need for washing steps that would otherwise dissociate weakly bound complexes. This wash‐free labeling approach enables the capture and identification not only of direct lactose‐binding proteins but also of their associated protein complex partners under mild, proximity‐labeling conditions.

These bead‐based studies highlight critical practical aspects for successful implementation in complex biological samples.

Proteins adsorbed on nanoparticle surfaces were investigated using nanoparticles co‐encapsulating biotin‐tyramide and chlorin e6 (Ce6). Upon 660 nm light irradiation, photoexcited Ce6 generates tyramide radicals from biotin‐tyramide, which label nanoparticle‐adsorbed proteins. The labeled proteins were then identified by LC‐MS/MS. This method achieves a temporal resolution of 5–300 s, enabling faster dynamic analysis of the protein corona compared to conventional methods [66].

Similarly, Zhang et al. developed a photocatalytic proximity labeling approach to identify proteins interacting with nanoplastic particles in living cells [76]. In this study, iodine‐substituted BODIPY (I‐BODIPY)‐NHS ester was covalently conjugated onto amino‐modified polystyrene nanoparticles as a photosensitizer. Upon white light irradiation, I‐BODIPY generates ROS that oxidize nearby proteins, enabling their covalent modification with a propargyl amine. The labeled proteins were then enriched and identified by LC‐MS/MS.

These nanoparticle‐based platforms demonstrate that photocatalytic labeling can be extended from purified proteins to complex biological mixtures, enabling proteome‐wide target identification through light‐triggered proximity labeling. By immobilizing photocatalysts on solid scaffolds, researchers achieve temporal control and capture weak interactions that would be difficult to detect by conventional methods. However, this approach sacrifices native cellular architecture, as proximity is defined relative to artificial scaffolds rather than physiological contexts. This regime represents a critical transition from fully controlled in vitro systems toward intact cellular environments.

### 3.3 Proximity Labeling on Cell

Photocatalytic proximity labeling on living cells represents a crucial bridge between chemical precision and biological relevance. Unlike in vitro or lysate‐based systems, on‐cell labeling preserves the native topology, membrane organization, and dynamic interactions that define cell‐surface biology. The plasma membrane is a crowded, heterogeneous interface where receptors, adhesion molecules, and signaling proteins assemble into transient nanoclusters and contact zones such as immune synapses. Capturing these microenvironments requires techniques that combine molecular selectivity with nanometer‐scale spatial resolution. Photocatalytic methods fulfill this need by using visible or red light to generate short‐lived reactive species—radicals, carbenes, nitrene, or ^1^O_2_—within confined diffusion distances, enabling site‐specific labeling of proteins in their physiological context. Through targeted installation of photocatalysts on antibodies, ligands, or membrane proteins, these systems allow temporal control, high spatial precision, and minimal perturbation to living cells. Sections 3.3.1–3.3.5 summarize recent advances in highly reactive species‐mediated strategies for cell‐surface labeling, their biological applications, and emerging challenges in quantifying proximity and selectivity on complex cellular interfaces.

#### 3.3.1 Radical‐Mediated Proximity Labeling on Cell

Hope and colleagues elucidated in detail the mechanism of protein labeling via tyramide radicals catalyzed by riboflavin tetraacetate (RFT). Building on this mechanistic insight, they applied the system to live‐cell labeling by targeting CD45 on Jurkat T cells through RFT‐conjugated antibodies. Upon blue‐light irradiation in the presence of biotin‐tyramide, selective enrichment of CD45‐proximal proteins was achieved, showing more than a twofold increase compared with controls lacking photocatalyst or light exposure [29]. Extending beyond single‐cell surface mapping, Oslund and colleagues developed photocatalytic cell tagging (PhoTag) technology specifically designed for interrogating cell–cell interactions [77]. Their approach employed single‐domain antibodies (VHHs) conjugated to photoactivatable flavin‐based cofactors, offering enhanced stability and tissue penetration compared to conventional antibodies. Following irradiation with visible light, the flavin photocatalyst generates tyramide radical tags that covalently modify proteins within a spatially restricted radius. Bechtel and colleagues further extended flavin‐photocatalyzed labeling to map protein environments at cell–cell interfaces [78]. By decorating antibodies and nanobodies targeting immunological synapse proteins with RFT, they performed light‐controlled labeling in co‐cultures of T cells and antigen‐presenting cells. The localized radical generation at intercellular contact zones allowed selective tagging of proteins within the immune synapse, yielding enrichment of canonical synaptic markers (CD3, LFA‐1, ICAM‐1) as well as unexpected proteins that may modulate synaptic adhesion and signaling.

**FIGURE 13:**
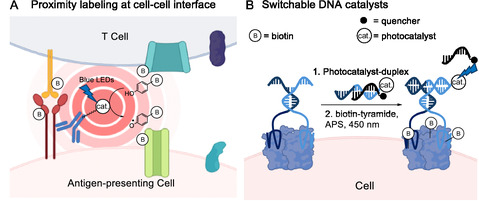
Representative tyramide radical‐mediated photocatalytic systems for cell‐surface proximity labeling.

Recent advances have explored programmable and conditional activation of photocatalytic proximity labeling, enabling on‐demand proteome mapping specifically at sites where both a protein of interest and a molecular trigger colocalize. Ogorek and colleagues developed switchable DNA catalysts that achieve stimulus‐responsive proximity labeling at protein–protein interaction sites [79]. Their system employs DNA secondary structures to control the proximity between a Ru(bpy)_2_(phen)‐type photocatalyst and quencher dye, effectively turning off photocatalysis until a molecular trigger induces conformational changes that separate these components. This DNA‐based switching mechanism allows the catalyst to activate only in response to specific chemical stimuli, enabling proteome mapping with unprecedented subcellular resolution at sites where both the protein of interest and the triggering molecule are present. In parallel, Zhou and Martell expanded this DNA‐directed approach to selectively activate photocatalytic reactions at cell–cell contact sites [80]. By coating two distinct cell populations with complementary DNA strands, they achieved site‐specific activation of Ru(bpy)_3_‐type photocatalysts specifically at intercellular interfaces where DNA hybridization occurs. This strategy demonstrated high spatial specificity for chemical labeling between contacting mammalian cells, offering a powerful tool to dissect the molecular composition of cell–cell communication zones without requiring genetic modification of target cells (Figure 13).

#### 3.3.2 Singlet‐Oxygen‐Mediated Labeling on Cell

^1^O_2_‐mediated photocatalysis represents a complementary approach to radical‐based labeling (see Section 2.2 for mechanisms). Through EnT from photoexcited catalysts to molecular oxygen, ^1^O_2_ preferentially oxidizes histidine residues, enabling orthogonal residue selectivity to radical‐mediated Tyr/Trp labeling. This proximity‐constrained reactivity, together with the ability to generate ^1^O_2_ upon visible‐ or red‐light excitation of photosensitizers such as halogenated xanthenes, porphyrins, or Ru(II) polypyridyl complexes, has positioned ^1^O_2_ chemistry as an attractive platform for spatially confined protein labeling, particularly through oxidation of histidine residues.

A recent study employed Metal–Organic Layers (MOLs), also known as two‐dimensional metal–organic nanosheets, incorporating TCPP to coat living cells and achieve ^1^O_2_‐mediated labeling directly at the cell surface [53]. This nanoPS‐PPL (nano‐photosensitizer proximity labeling) approach was successfully applied to comprehensive surfaceome profiling in living HeLa cells, identifying over 900 cell surface proteins with high specificity.

A mechanistic investigation reported by Qiu and colleagues systematically compared SET‐ and ^1^O_2_‐driven pathways in cell‐surface photocatalytic proximity labeling. In this study, Ru(bpy)_3_‐based photocatalysts were evaluated under identical illumination conditions using two representative substrates: a tyramide probe for SET‐mediated tyramide radical formation targeting tyrosine residues, and a hydrazide probe for ^1^O_2_‐mediated oxidation of histidine residues. Quantitative proteomic analysis revealed that the ^1^O_2_‐driven hydrazide labeling pathway afforded higher labeling efficiency and greater site specificity than the SET‐based tyramide system [81].

In another significant contribution, Müller and colleagues developed a versatile ^1^O_2_–mediated photocatalytic labeling strategy termed LUX‐MS (light‐controlled proximity labeling and mass spectrometry), in which a thiorhodamine‐based small‐molecule photosensitizer was conjugated to diverse biological ligands to enable light‐triggered labeling of nearby proteins on living cell surfaces [82]. This platform demonstrated remarkable flexibility by coupling the thiorhodamine catalyst to monoclonal antibodies (e.g., anti‐CD20 and anti‐EGFR), small‐molecule ligands (such as the cardiac glycoside CG1), and even viral particles and bacteriophages. Upon visible‐light illumination, localized generation of ^1^O_2_ enabled oxidation and biotinylation of proximal proteins without the need for genetic modification.

#### 3.3.3 μMap Applications on Cell Surface

**FIGURE 14:**
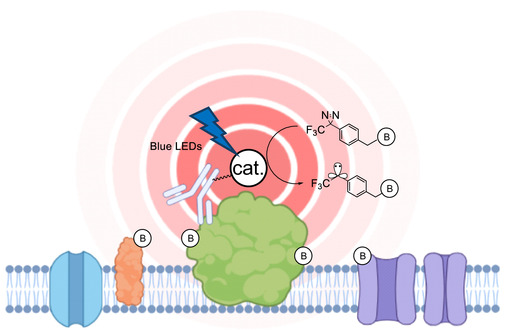
Concept of μMap showing Dexter energy transfer mechanism and carbene generation.

The development of microenvironment mapping (μMap) by Geri and colleagues in 2020 represented a transformative advance in photocatalytic proximity labeling (Figure 14) [44]. The application of μMap to immune checkpoint molecules has provided crucial insights into the molecular mechanisms underlying cancer immunotherapy. When Geri and colleagues targeted PD‐L1 on human B cells using an antibody‐iridium conjugate system, they discovered previously unknown interactions with CD30 and CD300A, proteins that showed >10‐fold enrichment in the PD‐L1 microenvironment. This discovery suggested the existence of immune checkpoint crosstalk pathways beyond the canonical PD‐1/PD‐L1 axis, with potential implications for combination therapy development. The technique's ability to perform trans‐labeling across immune synapses further demonstrated its utility in studying cell–cell interactions, revealing the dynamic nature of immune checkpoint signaling at the interface between T cells and antigen‐presenting cells.

Beyond immunomodulatory targets, μMap has been applied to oncogenic receptor systems. Extending μMap from basic interactome mapping to clinically relevant systems, the study by Knutson and colleagues performed micromapping of HER2 in breast‐cancer cell lines. The workflow utilized iridium‐conjugated antibodies targeting HER2, followed by blue‐light activation to label proximate proteins, thereby identifying previously unrecognized resistance‐modulating interactors such as galectins and PTPRF [83]. Their results link surface‐proximal molecular architectures with targeted‐therapy responsiveness, exemplifying how μMap can bridge high‐resolution mapping with therapeutic insight in cancer biology.

The COVID‐19 pandemic highlighted the critical need for understanding virus‐host interactions at molecular resolution. Multiple research groups rapidly deployed μMap to investigate SARS‐CoV‐2 spike protein interactions with host cell surfaces, yielding complementary insights into viral entry mechanisms. Suzuki and colleagues conjugated iridium catalysts directly to recombinant Wuhan spike proteins and identified four novel entry factors—NRP2, EPHA7, SLC6A15, and MAL2—in Calu‐3 lung epithelial cells, with co‐expression of these factors alongside ACE2 enhancing viral uptake by 5–7‐fold [84]. In parallel work, Datta and colleagues discovered PTGFRN and EFNB1 as additional entry facilitators, with knockdown experiments confirming their functional relevance through 30%–45% reduction in viral infection [85]. These findings collectively revealed the complex landscape of SARS‐CoV‐2 entry factors beyond ACE2, providing potential therapeutic targets for COVID‐19 intervention.

The integration of metabolic labeling with μMap technology has enabled unprecedented insights into glycosylation‐dependent protein interactions at the cell surface. Meyer and colleagues developed GlycoMap, which combines metabolic incorporation of azido‐modified sialic acids with strain‐promoted azide–alkyne cycloaddition (SPAAC) chemistry to site‐specifically introduce iridium catalysts at glycosylated positions [86]. This approach revealed striking differences between highly sialylated cancer cells and normal cervical cells, with 14 solute carrier family proteins identified as sialylation‐dependent interactors.

The versatility of μMap extends to studying dynamic receptor processes such as dimerization and clustering. Recent developments include split‐μMap systems where split intein‐mediated protein trans‐splicing reconstitutes the functional photocatalyst only upon receptor dimerization—the catalyst fragments remain inactive when attached to monomeric receptors but spontaneously splice together to form active catalyst when receptors dimerize, enabling selective labeling of activated receptor complexes. Time‐resolved experiments with 15‐min intervals have captured the temporal dynamics of EGFR dimerization upon ligand stimulation, revealing transient interaction partners that would be missed by endpoint analyses. Advanced implementations have demonstrated spatiotemporal resolution sufficient to distinguish between monomeric and dimeric receptor states, with applications in understanding ligand‐induced conformational changes and downstream signaling cascades [87].

Complementarily, the μMap‐Interface strategy expanded the technique to dynamic cell–particle contacts, enabling time‐resolved photocatalytic labeling of phagocytic interfaces [88]. Using iridium‐coated IgG beads and synchronized phagocytosis, this approach revealed temporal remodeling of the macrophage surfaceome and uncovered F11R (JAM‐A) as a negative regulator of particle uptake. These findings highlight the growing capability of μMap platforms to probe transient, process‐specific protein interactions at subcellular interfaces.

Expanding the reach of photocatalytic proximity labeling beyond μMap systems, Lin and colleagues recently introduced a complementary platform termed MultiMap, which enables multiscale labeling of protein neighborhoods on and between cells [50]. Using a single, commercially available photocatalyst—Eosin Y—conjugated to antibodies, they demonstrated that three distinct reactive probes (diazirine, aryl azide, and tyramide) could be used to generate carbene, nitrene, and tyramide radical intermediates, respectively, thereby achieving tunable labeling ranges from sub‐10 nm to several tens of nanometers. This modular design allowed controlled mapping of the cell‐surface interactome under varying distance constraints without altering the photocatalyst itself. When applied to EGFR‐expressing epithelial cells, MultiMap identified both proximal binding partners such as integrins and more distal co‐localized membrane components, while competition with EGF confirmed labeling specificity. Notably, by extending the same strategy to cell–cell contact interfaces, the authors captured intercellular protein neighborhoods within T cell–tumor synapses, demonstrating the feasibility of photocatalytic labeling across biological membranes. The ability to modulate labeling distance through probe chemistry rather than protein tether length represents a conceptual advance that complements μMap's nanometer‐scale precision, providing a versatile framework for dissecting protein interactions across multiple spatial regimes of the cell surface (Table 5).

**TABLE 5:** On‐cell μMap proximity labeling and its applications.

References	Catalyst structure	Target protein	Catalyst installation	Labeling reagent
Gerl et al., [44]	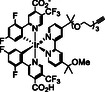	PD‐L1 (also CD45, CD47, CD29)	Lys mod. with NHS‐azide and CuAAC	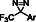
Datta et al. [85]	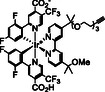	SARS‐CoV‐2 spike protein	Lys mod. with NHS‐azide and CuAAC	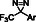
Suzuki et al. [84]	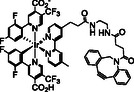	SARS‐CoV‐2 spike protein	Lys mod. with NHS‐azide and SPAAC	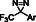
Meyer et al. [86]	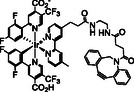	Sialylated cell‐surface glycoproteins	Metabolic incorporation of (AC_4_ManNAz), then SPAAC	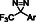
Knutson et al. [83]	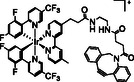	HER2	Lys mod. with NHS‐azide and SPAAC	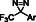
Kofoed et al. [87]	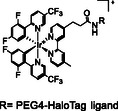	EGFR and other cell‐surface receptors	Genetic fusion of caged split SpyTag/HaloTag	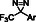
Huth et al. [88]	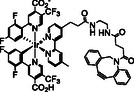	IgG‐opsonized polystyrene beads	Lys mod. of IgG on beads with NHS‐azide then SPAAC	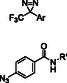
Lin et al. [50]	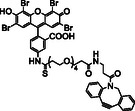	EGFR and cell–cell contact interfaces	Lys mod. of IgG on beads with NHS‐azide then SPAAC	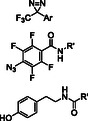

Despite its transformative capabilities, μMap faces several technical challenges that warrant continued development. The multi‐step synthesis of iridium photocatalysts remains complex, and these catalysts often exhibit limited stability in solution, making their preparation and handling more demanding than those of other photocatalysts. Prolonged illumination beyond 30 min can generate ROS leading to phototoxicity, although this is rarely problematic given the typical 10–30 min labeling times. Non‐specific accumulation of cationic iridium complexes in mitochondria due to membrane potential can create background signal, an issue that will be discussed in detail in the later section.

#### 3.3.4 Chromatin Interactome Mapping in Isolated Nuclei

While the µMap applications described above focused on extracellular protein domains, the technology has been successfully extended to nuclear proteins using isolated nuclei as an intermediate experimental system. This approach bridges extracellular labeling with true intracellular applications by providing a controlled environment for studying chromatin‐associated protein interactions.

Building on photocatalytic proximity labeling approaches, Seath et al. developed a µMap method specifically designed for mapping protein–protein interactions in the nuclear environment. In this system, Ir photocatalysts are site‐specifically incorporated onto target proteins through engineered split intein‐mediated protein trans‐splicing, enabling traceless catalyst installation [89]. Upon blue light irradiation, the iridium catalyst activates diazirine‐biotin probes through DET, generating reactive carbenes that covalently label proteins within 4 nm radius. This exceptionally short labeling distance allows µMap to detect subtle perturbations in chromatin interactomes caused by single‐amino‐acid mutations or small‐molecule inhibitors.

Expanding upon the µMap platform, Douglas and colleagues developed MesoMap, a method that enables proximity labeling across the entire chromatin landscape rather than at a single protein target [90]. By incorporating iridium photocatalysts onto multiple histone variants (H2A, H3.1, H3.3, and H4) simultaneously, MesoMap captures system‐wide changes in chromatin occupancy in response to cellular stimuli.

#### 3.3.5 Labeling Radius Measurement

The precise determination of labeling radius represents a fundamental parameter for understanding the spatial resolution capabilities of proximity labeling technologies. Despite the critical importance of this metric for interpreting labeling results and comparing methodological approaches, only a few studies have systematically quantified the effective labeling distances achieved by various systems.

Before the advent of photocatalytic approaches, several non‐photocatalytic proximity labeling methods had already addressed this concept through diverse chemistries. For instance, EMARS [91] employed HRP‐conjugated antibodies to generate nitrene intermediates, where both radical diffusion and the physical reach of antibody tethers defined the labeling spread. BioID introduced an in vivo molecular ruler using the Nup107–160 Y‐complex, revealing an effective labeling radius of ∼10 nm [61], while TurboID was shown to be effective over a somewhat broader range of up to ∼35 nm [62]. In contrast, peroxidase‐based tyramide labeling exhibits a much broader diffusion profile, with recent sub‐organelle proteomic mapping demonstrating tyramide radical propagation of roughly 200 nm [63]. DNA origami‐based measurements have also been utilized to profile labeling distances with nanometer precision, providing mechanistic insights into the spatial constraints of various labeling enzymes [92].

Oakley and colleagues established rigorous methodological frameworks for measuring labeling radii in photocatalytic proximity labeling systems [64]. Using a reductionist model with dual‐antibody separation on BSA‐coated coverslips combined with super‐resolution microscopy, they directly visualized spatial labeling extent through biotinylation patterns, reporting measurements as full width at half maximum (FWHM) values—a standardized metric describing distribution width at 50% peak intensity.

Their systematic analysis revealed striking differences between systems: the μMap carbene‐based platform exhibited a FWHM of 54 ± 12 nm, while peroxidase‐based tyramide radical labeling showed 269 ± 41 nm—a fivefold difference. Critically, control experiments using biotinylated secondary antibodies alone revealed clusters of 57 ± 12 nm, nearly identical to μMap measurements. This elegant finding demonstrated that the observed μMap labeling radius is primarily limited by the physical dimensions of the antibody tether rather than carbene diffusion. Given that carbenes have a 2‐nanosecond half‐life predicting ∼4 nm theoretical diffusion distance, the true reactive radius is indeed in the low‐nanometer range as expected. After accounting for the antibody assembly contribution (∼57 nm), the ∼200 nm difference between carbene and tyramide radical labeling represents a dramatic disparity in spatial resolution, highlighting that HRP‐generated radicals diffuse over vastly greater distances, fundamentally limiting peroxidase‐based proximity labeling precision.

Oakley's work also explored alternative reactive species generated through photocatalysis. When utilizing phenyl azide probes activated through SET, they observed dramatically different labeling patterns. The standard phenyl azide probe (Biotin‐PEG3‐PhN_3_) yielded a FWHM of 119 ± 33 nm, while the tetrafluorinated analog (Biotin‐PEG3‐PhF_4_N_3_) showed improved spatial resolution with a FWHM of 68 ± 17 nm. Furthermore, they demonstrated that extending the PEG linker length from PEG3 to PEG24 reduced the diffusion coefficient approximately twofold, resulting in a tuned intermediate labeling radius of 80 ± 28 nm—providing a simple molecular strategy for customizing spatial resolution through diffusion control. These measurements demonstrate that the choice of reactive intermediate profoundly influences spatial resolution, with nitrene‐based systems occupying an intermediate position between carbene and radical‐based approaches.

Building upon these foundational measurements, Bartholow and colleagues employed an orthogonal approach using site‐specific catalyst conjugation directly onto primary antibodies to eliminate the secondary antibody contribution to labeling distance [65]. Using the well‐characterized trastuzumab‐HER2 complex as a model system, they engineered eight distinct single‐site iridium catalyst attachment points across the Fab fragment. Through comprehensive mass spectrometry analysis of intramolecular and intermolecular labeling patterns, they determined that carbene‐mediated labeling reached a maximum distance of approximately 110 Å (11 nm) from the catalyst site. This measurement represents the absolute maximum labeling distance rather than a FWHM value, providing complementary information about the spatial extent of carbene reactivity.

#### 3.3.6 Controlling Proximity Labeling with Red Light

The development of red‐shifted photocatalytic systems represents a transformative advancement in proximity labeling technology, addressing the critical limitation of tissue penetration that has historically confined photocatalytic methods to cultured cell applications. While blue light (450 nm) penetrates biological tissues to depths of approximately 1 mm, its use also presents a significant drawback: it can activate endogenous cellular photosensitizers such as flavins and porphyrins, generating ROS that cause undesired background labeling and phototoxicity [11]. In contrast, deep‐red (650–700 nm) and NIR wavelengths (700–800 nm) can reach 5–10 mm [93], opening unprecedented opportunities for proteomic analysis in complex biological environments including organoids, tissue sections, and ultimately living organisms.

The pioneering μMap‐Red platform developed by Buksh and colleagues established the feasibility of red‐light photocatalytic proximity labeling using Sn(IV) chlorin e6 catalysts [23]. This system exploits NADH‐mediated reductive quenching to activate phenyl azide probes under 660 nm illumination, generating aminyl radicals through SET mechanisms. Critically, the platform enabled direct labeling in whole mouse blood—a complex biological matrix previously inaccessible to photocatalytic approaches—achieving comprehensive profiling of erythrocyte surface proteins with minimal sample manipulation.

Building upon the concept of long‐wavelength activation, Tay and colleagues introduced Os(II) photocatalysts that respond to deep‐red light (660 nm) for targeted protein labeling in localized environments [94]. Their system generates triplet nitrenes through SET mechanisms, offering complementary reactivity to carbene‐based approaches. When applied to three‐dimensional spheroids and tumor tissue sections, the osmium catalysts successfully labeled EpCAM and identified CDH3 as a co‐expressed protein with significant clinical relevance. The ability to perform proximity labeling in tissue‐like structures demonstrated the potential for studying protein interactions in their native architectural context, preserving spatial relationships lost in conventional cell culture systems.

Ryu and colleagues developed TTMAPP and n‐Pr‐DMQA^+^ photocatalysts capable of generating fluoroalkyl radicals for protein functionalization under 660 nm illumination [31]. Significantly, leveraging the superior tissue penetrance of deep‐red light, they demonstrated photocatalytic protein fluoroalkylation within intact tissue specimens.

The recent advancement in deep‐red photocatalytic proximity labeling comes from Lou and colleagues, who developed CAT‐Tissue, an ultrafast labeling platform specifically designed for dissecting tumor‐immune interactions in primary tissues [54]. This innovative approach combines nanobody‐chlorin e6 photocatalysts with biotin‐aniline probes to achieve rapid and comprehensive detection of cell–cell interactions in both co‐culture systems and primary tumor sections.

Complementing these single‐probe approaches, Tong and colleagues developed a multiprobe photoproximity labeling platform that utilizes a single chlorin e6 catalyst with two distinct reactive probe types under red light excitation [95]. In addition to a carbene‐based pathway that employs sulfonium diazo probes to capture the EGFR interactome in glioblastoma cells, the same photocatalyst can also generate ^1^O_2_ to drive an aniline‐based labeling chemistry, enabling a complementary SOG‐mediated mode of probe activation. Together, these dual chemistries demonstrate that probe diversification can substantially expand proteome coverage while preserving the advantages of red‐light activation (Table 6).

**TABLE 6:** Comparison of red‐light photocatalytic proximity labeling platforms.

Reference	Catalyst structure	Target protein	Light wavelength, nm	Labeling reagent
Buksh et al. [23]	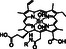	Whole blood labeling	660	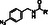
Tay et al. [94]	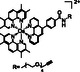	3D spheroids, tumor sections	660	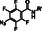
Ryu et al. [31]	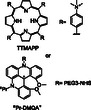	Intact tissue labeling	660	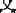
Lou et al. [54]	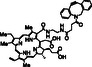	Tumor‐immune profiling	660	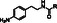
Tong et al. [95]	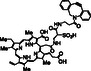	EGFR in glioblastoma	660	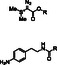

### 3.4 Proximity Labeling in Cell

Extending photocatalytic proximity labeling from the cell surface into the intracellular space represents a profound shift in both chemical strategy and biological scope. While cell‐surface labeling benefits from the extracellular accessibility of targets and the straightforward installation of photocatalysts via antibodies or ligands, the intracellular environment presents a fundamentally more complex landscape. Here, proteins reside within highly compartmentalized, redox‐active, and crowded microenvironments—the cytosol, nucleus, mitochondria, endoplasmic reticulum, lysosomes, and other organelles—each characterized by distinct pH, ionic strength, oxygen tension, and metabolite compositions.

Critically, two interrelated biophysical parameters profoundly influence the operational characteristics of photocatalytic proximity labeling in these spaces. First, the high concentration of cellular reductants such as glutathione (1–10 mM in the cytosol) and NADH poses intrinsic chemical challenges by rapidly quenching reactive intermediates, effectively reducing their diffusion distance and lifetime [96, 97]. Second, macromolecular crowding and viscoelasticity differ substantially between compartments—the cytosol exhibits an effective viscosity several‐fold higher than aqueous buffer, while organelle lumina (endoplasmic reticulum (ER), mitochondrial matrix) and the extracellular matrix each present distinct microviscosity profiles [98, 99, 100, 101]. These parameters modulate not only the diffusion coefficients of short‐lived reactive species such as radicals, carbenes, and ^1^O_2_, but also the conformational freedom of flexible antibody or linker tethers used to position photocatalysts, effectively shifting the operational labeling radius in a compartment‐specific manner. Successfully mapping protein interactions within these spaces therefore demands not only membrane‐permeable photocatalysts but also strategies to control subcellular localization, minimize phototoxicity from prolonged illumination, and optimize the choice of reactive intermediate—carbene, nitrene, radical, or ^1^O_2_—considering both labeling efficiency and amino acid selectivity within densely packed intracellular proteomes.

The intracellular application of photocatalytic proximity labeling has evolved through two complementary strategic frameworks. The first approach, discussed in Section 3.4.1, employs freely diffusible small‐molecule photocatalysts that spontaneously penetrate cellular membranes and, in many cases, accumulate preferentially within specific organelles due to their physicochemical properties. Before turning to the targeted photocatalytic systems, Section 3.4.2 introduces aggregate profiling as an intermediate conceptual step. This section highlights how photocatalytic labeling can be applied to characterize mesoscale protein assemblies without the need for genetically installed targeting motifs, thereby bridging the gap between untargeted free‐catalyst strategies and the highly localized methods that follow. Sections 3.4.3 through 3.4.7 present a second, more spatially precise framework encompassing a range of targeted photocatalytic strategies. These include organelle‐directed and affinity ligand–directed approaches, tag‐protein–mediated µMap systems, HaloTag‐based intracellular methods using non‐iridium catalysts, and fully genetically encoded photocatalytic labeling platforms. Together, these methods demonstrate how photocatalytic activity can be directed to defined subcellular locales with increasing specificity, offering a versatile toolkit for high‐resolution proximity labeling.

#### 3.4.1 Free Catalyst Approach

The most conceptually straightforward strategy for intracellular photocatalytic proximity labeling involves the direct introduction of membrane‐permeable small‐molecule photocatalysts into living cells. Unlike antibody‐ or genetic‐fusion‐based approaches, this “free catalyst” method requires no prior modification of target proteins, no genetic engineering, and no tedious chemical synthesis of ligand‐conjugated photocatalysts. Instead, cells are simply incubated with lipophilic photocatalytic compounds that spontaneously cross plasma membranes, distribute throughout the cytoplasm and organelles, and—upon light activation—generate reactive intermediates capable of covalently modifying nearby proteins. Conceptually, free catalyst approaches can be understood along two orthogonal dimensions: (i) intracellular distribution, which is governed primarily by the physicochemical properties of the photocatalyst, and (ii) labeling selectivity, which is dictated by the nature of the photogenerated reactive intermediates. Depending on how these two parameters are balanced, free catalyst strategies can range from global, proteome‐wide labeling to organelle‐confined and even multi‐omics profiling.

At one extreme of this spectrum, free catalysts distribute broadly throughout the cell without intentional subcellular targeting, enabling proteome‐wide interrogation of reactive amino acid chemistry. In this context, Zhai and colleagues developed an orthogonal intracellular labeling strategy exploiting ^1^O_2_ generation for histidine‐selective protein labeling in living cells [102]. Through systematic screening of nucleophilic trapping reagents, the authors identified 3‐ethynylaniline (3‐EA) as the optimal probe, providing superior labeling efficiency compared to conventional amine nucleophiles while enabling alkyne handle installation for subsequent bioorthogonal conjugation. This ^1^O_2_‐mediated approach proved particularly powerful for profiling metal‐binding proteomes, as histidine residues frequently coordinate metal ions in metalloproteins. The method successfully identified hundreds of metal‐binding proteins in HeLa cells, revealing both known metalloproteins and previously uncharacterized metal‐interacting candidates.

While such untargeted strategies emphasize reaction selectivity over spatial control, a complementary and widely exploited paradigm leverages the intrinsic subcellular accumulation behavior of small‐molecule photocatalysts to achieve organelle‐specific proximity labeling. Among intracellular organelles, mitochondria have emerged as a particularly tractable target for free catalyst approaches. The preferential accumulation of cationic lipophilic molecules in mitochondria has long been recognized as both a challenge and an opportunity in chemical biology. Driven by the large negative membrane potential (approximately −180 mV) across the inner mitochondrial membrane, positively charged lipophilic compounds can accumulate several hundred‐fold within the mitochondrial matrix through the Nernst equation. This phenomenon, initially exploited for mitochondria‐selective fluorescent imaging probes, has been elegantly repurposed for proximity labeling applications.

Building upon this foundational principle, several research groups have systematically explored the application of free catalyst approaches for mitochondrial proximity labeling. Wang and colleagues pioneered the use of organic dyes—acridine orange, fluorescein, and rhodamine 123—as biocompatible photocatalysts for protein labeling with aryl azides, demonstrating that rhodamine 123 could selectively label mitochondrial proteins through its inherent organellar localization [51]. This photocatalytic azide conjugation approach yielded benzazirines and ketenimines from triplet nitrenes for protein nucleophilic residue trapping, establishing a paradigm for metal‐free intracellular photocatalytic labeling.

Subsequent developments have focused on expanding the chemical toolkit and improving labeling efficiency. Huang et al. introduced the CAT‐Prox (bioorthogonal and photocatalytic decaging‐enabled proximity labeling) strategy, using Ir[dF(CF_3_)ppy]_2_(dtbbpy) as a bioorthogonal and mitochondria‐targeting catalyst that enabled photocontrolled, rapid rescue of azidobenzyl‐caged quinone methide for proximity‐based protein labeling, achieving approximately 70% mitochondrial specificity from up to 300 enriched proteins [67]. This approach successfully profiled mitochondrial proteomes in both easily transfectable HeLa cells and hard‐to‐transfect macrophage RAW264.7 cells, demonstrating the versatility of photocatalytic methods across diverse cellular contexts.

Building upon the CAT‐Prox system, the same research group subsequently developed CAT‐S, a significantly enhanced version that addressed the limitations of the original approach. CAT‐S introduced thio‐quinone methide (thioQM) as a novel labeling warhead with higher reactivity than conventional QM probes, attributed to the lower electronegativity of sulfur and the increased stability of the reactive zwitterionic resonance due to the larger atomic radius of sulfur [103]. This enhanced reactivity, combined with the thioQM's preference for reacting with soft nucleophiles in an aqueous medium, enabled the system to extend proximity labeling to primary living samples.

Expanding beyond protein labeling, the same photocatalytic decaging chemistry was adapted for RNA profiling through the development of CAT‐seq, a bioorthogonal photocatalytic labeling and sequencing strategy that enables high‐resolution, in situ profiling of mitochondrial RNA without genetic manipulation [104]. CAT‐seq employed an optimized QM probe specifically for efficient RNA labeling, with light‐driven activation enabling spatiotemporally controlled covalent attachment to RNA molecules within the mitochondrial matrix. The key to this orthogonal system lies in the differential reactivity: QM probes preferentially react with cytidine residues in RNA through hydrogen‐bond‐assisted conjugation, while thioQM probes exhibit enhanced selectivity toward nucleophilic amino acid residues in proteins. Most remarkably, by leveraging the distinctive chemistry of quinone methide warheads, the team established an orthogonal labeling system enabling synchronous RNA and protein multi‐omics profiling within the same sample, providing a holistic view of mitochondrial molecular landscapes by linking transcriptomic and proteomic information in living cells. This evolution from CAT‐Prox through CAT‐S to CAT‐seq represents a progression from proof‐of‐concept protein labeling to comprehensive multi‐omics analysis in clinically relevant primary samples.

Recent advances have addressed critical limitations of visible light‐based approaches by shifting toward longer wavelengths. Zeng and colleagues developed the proximity labeling strategy using near‐infrared excitation (PL‐NIR), utilizing IR780's NIR absorption and mitochondrial targeting properties combined with 3‐ethynylaniline (3‐EA) as the nucleophilic trapping reagent to selectively label up to 245 mitochondrial proteins in living HeLa cells [55]. This NIR‐based strategy avoids absorption overlap with endogenous chromophores and reduces light scattering in biological samples, significantly improving labeling efficiency and identification coverage.

Beyond protein proximity labeling, photocatalytic strategies have also been adapted to capture transient RNA‐protein interactions. Luo and colleagues demonstrated that eosin Y, a widely available organic photosensitizer, could mediate direct crosslinking between RNA and proteins in living cells upon visible light irradiation [105]. This approach exploits eosin Y's ability to generate reactive species that simultaneously modify both nucleic acids and proteins, covalently capturing RNA‐protein complexes in their native cellular context.

Most recently, CAT‐ER was introduced as a nongenetic ER proteomics system providing in situ labeling, spatiotemporal resolution, and compatibility across diverse cell types by combining an ER‐targeted iridium photocatalyst with a thioOM probe [106]. CAT‐ER achieved high specificity in enriching ER proteins comparable to enzymatic methods while enabling use in hard‐to‐transfect cell types, including HeLa and immune cells (Raji, Jurkat, RAW264.7), and enabling time‐resolved profiling of ER proteome dynamics during stress‐induced apoptosis, uncovering the molecular transitions underlying UPR‐to‐apoptosis progression.

#### 3.4.2 Aggregate Profiling

While the free catalyst approaches described in the previous section exploit the intrinsic organellar accumulation of photocatalysts, a conceptually related but functionally distinct application involves photocatalysts engineered to selectively bind protein aggregates. Protein aggregation is a hallmark of numerous neurodegenerative diseases, including Alzheimer's disease (characterized by amyloid‐β plaques and tau tangles), Parkinson's disease (α‐synuclein aggregates), and Huntington's disease (huntingtin aggregates). Understanding the molecular composition of the microenvironment surrounding these pathological aggregates—the aggregate‐proximal proteome—is critical for elucidating disease mechanisms, identifying potential therapeutic targets, and developing diagnostic biomarkers. However, traditional biochemical fractionation methods struggle to distinguish proteins genuinely associated with aggregates from those merely co‐precipitating during sample preparation, while immunofluorescence‐based colocalization studies lack the resolution and throughput necessary for comprehensive proteomic analysis. Photocatalytic proximity labeling offers an elegant solution: by conjugating photocatalysts to aggregate‐binding moieties, researchers can achieve selective enrichment at disease‐relevant protein deposits, followed by light‐triggered labeling of the surrounding proteome in situ.

This strategy was pioneered by Feng and colleagues, who developed a cell‐permeable diacetyl derivative of Rose Bengal for selective photoinduced proximity labeling and crosslinking of aggregated proteome [107]. This work established the first photoinduced proximity labeling method capable of simultaneously profiling aggregated proteome composition and interaction networks in living cells.

Complementing this approach, Sun and colleagues developed ultra‐photosensitized fluorescent protein chromophore mimics, achieving over 1000‐fold enhancement in ROS production compared to natural KillerRed [108]. Through a 12 × 12 combinatorial library of synthetic FP chromophore analogs, they discovered that chromophores with larger dipole moments exhibited higher ROS yields, with the optimal probe A7B3 demonstrating superior photocatalytic activity. Mechanistic studies revealed Type I photosensitization via superoxide anions and hydroxyl radicals (without ^1^O_2_ production), providing expanded reactivity toward diverse protein residues (His, Glu, Phe, and Trp) using propargyl amine. Biophysical analysis revealed that the captured interactome exhibited increased disorder propensity (DisProt rank) and aggregation propensity (Aggrescan3D score), confirming intrinsic phase separation tendency. Extending aggregate‐targeted photocatalysis to pathological tissue deposits, Sun and colleagues developed AmyCAT, in which an amyloid‐binding fluorescent protein chromophore‐derived probe (A2B1) was paired with a diazo substrate (S3) to achieve Dexter energy transfer (DET)‐driven, spatially confined proximity labeling of amyloid plaques in Alzheimer's disease mouse brain sections. This strategy enabled direct enrichment and LC–MS/MS profiling of 44 enriched plaque‐associated proteins, recovering canonical AD biomarkers such as APP, APOE, and GFAP together with additional chaperone‐, immune/inflammatory‐, and cytoskeleton‐related proteins, including CLU, HSPB1, TAPBP, C4B, FGA, SERPINA3N, Vim, and FLNC. These hits were further validated by colocalization with amyloid deposits, indicating that AmyCAT can dissect disease‐relevant molecular neighborhoods in situ with higher enrichment of key deposition biomarkers than conventional homogenization‐ or LCM‐based workflows [109]. Together, these aggregate‐targeted photocatalytic strategies—combining selective binding, efficient photosensitization, and crosslinking capabilities—represent powerful approaches for dissecting molecular ecosystems surrounding protein aggregates in neurodegenerative diseases and phase‐separated condensates.

#### 3.4.3 Organelle‐Targeted Ligand‐Directed Photocatalytic Proximity Labeling

Beyond intrinsic accumulation properties, photocatalytic proximity labeling can be strategically directed to specific organelles by conjugating photocatalysts with organelle‐targeting moieties, enabling systematic profiling of compartment‐specific proteomes without genetic manipulation. This ligand‐directed approach combines the spatiotemporal control of photocatalysis with the subcellular precision of chemical targeting, offering a powerful alternative to enzyme‐based methods, particularly in hard‐to‐transfect cell types.

While Section 3.4.1 described free catalysts that passively accumulate in mitochondria via their intrinsic lipophilic cationic properties, active targeting strategies using dedicated mitochondrial‐localizing moieties offer enhanced specificity and generalizability. Pioneering this approach, researchers developed a photo‐oxidation driven proximity labeling strategy employing organelle‐localizable organic photoactivated probes equipped with triphenylphosphonium (TPP)—a well‐established mitochondrial targeting group—that generate reactive species upon light activation to selectively label mitochondrial proteins in their native environment [49]. Integration of this photocatalytic proximity labeling with quantitative proteomics enabled profiling of the mitochondrial proteome with up to 310 mitochondrial proteins identified at 64% specificity in HeLa cells, and successfully deciphered mitochondrial proteome dynamics in hard‐to‐transfect drug‐resistant Huh7 and LPS‐stimulated HMC3 cells, quantifying differential proteins intimately linked to drug resistance and neuroinflammation. This organelle‐targeted strategy has also been extended beyond proteome analysis to subcellular transcriptome profiling. Liang and colleagues compared four mitochondria‐localizable AIE photosensitizers and identified TFPy as the optimal probe for RNA proximity labeling, enabling the capture of mitochondria‐associated transcripts, including mitochondria‐encoded RNAs as well as nuclear‐derived RNAs localized near the outer mitochondrial membrane and interacting organelles [110]. Extending organelle‐targeted photocatalytic proximity labeling to the nucleus required strategies to concentrate photocatalysts within chromatin microenvironments. Li and colleagues employed a nucleus‐localized ^1^O_2_ generator using dibromofluorescein (DBF) conjugated to Hoechst 33 342 (a DNA minor groove binder) to label chromatin‐associated biomolecules through spatially‐restricted oxidation under visible light, with fluorescence confocal microscopy, Western blot, and RT‐qPCR confirming desirable spatial resolution for enriching chromatin‐associated RNAs and proteins [111]. In parallel, Tamura and colleagues developed a conceptually similar photoactivatable proximity labeling method employing the same DBF‐Hoechst scaffold, which upon green light irradiation generates ^1^O_2_ that reacts with an o‐phenylenediamine (PDA)‐based biotin probe to promiscuously tag nearby proteins [112]. Their systematic characterization revealed that DBF‐Hoechst exhibited enhanced fluorescence and doubled ^1^O_2_ generation upon DNA binding, providing a “turn‐on” mechanism that suppressed false‐positive labeling from unbound photocatalyst, while the acetylated derivative AcDBF‐Hoechst achieved cell permeability through esterase‐mediated activation. Li and colleagues reported FAP‐seq, in which a genetically encoded fluorogen‐activating protein (FAP) localizes the probe to defined intracellular regions, where FAP‐bound MG‐HI enables wash‐free, near‐infrared‐light‐induced RNA proximity labeling and transcriptome analysis. MG‐HI was designed as a monoiodinated malachite green derivative based on the FAP–MG fluorogenic system, in which FAP binding restricts dye rotation to activate fluorescence, while the heavy‐atom substitution enhances singlet oxygen generation for efficient proximity labeling [113].

Addressing the challenge of profiling lysosomes—whose acidic environment complicates conventional chemical labeling strategies—Zhang and colleagues developed CAT‐Lyso, combining a lysosome‐targeting photocatalyst with a thioQM probe to enable in situ lysosomal proteomics without genetic manipulation [114]. CAT‐Lyso successfully profiled lysosomal proteomes across diverse cell lines, including hard‐to‐transfect macrophages (RAW264.7) and B lymphocytes (Raji), identifying cell type‐specific patterns and uncovering previously unrecognized lysosomal proteins.

Guo and colleagues developed LipoID, a small‐molecule photocatalytic method for in situ profiling of the LD‐interacting proteome. Employing an LD‐targeting photosensitizer selected from an X–furan–Y probe panel, LipoID enabled proximity‐dependent labeling and enrichment of LD‐associated proteins in living cells without genetic fusion to LD marker proteins. Proteomic analysis identified canonical LD proteins such as PLIN2, PLIN3, LDAH, and LSS, while also capturing 163 lipid‐related proteins and uncovering additional LD‐proximal candidates including CLINT1 and SCP2. Notably, the resulting interactome pointed to extensive tethered interorganelle contacts, particularly between LDs and mitochondria, and comparative analysis further highlighted VDAC3 as a potential regulator of LD–mitochondria proximity through PLIN3‐associated interactions. These findings were complemented by metabolomic changes consistent with remodeling of lipid metabolism downstream of LD–mitochondria coupling [115].

Together, these ligand‐directed photocatalytic proximity labeling strategies demonstrate a versatile platform for systematic organellar proteomics, offering genetic manipulation‐free alternatives to enzyme‐based methods with broad applicability across cell types and subcellular compartments (Table 7).

**TABLE 7:** Proximity labeling using photocatalysts localized to specific organelles.

References	Catalyst structure	Targeted cellular component	Reactive species or labeling probe
Wang et al. [51]	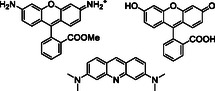	Mitochondria	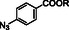
Huang et al. [67]	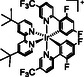	Mitochondria	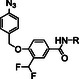
Liu et al. [103]	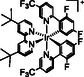	Mitochondria	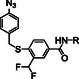
Bi et al. [104]	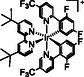	Mitochondria	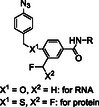
Zeng et al. [55]	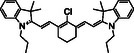	Mitochondria	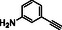
Wang et al. [49]	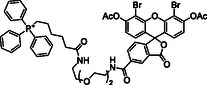	Mitochondria	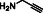
Li et al. [111]	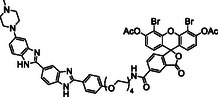	Nucleus	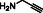
Tamura et al. [112]	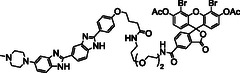	Nucleus	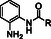
Zhang et al. [114]	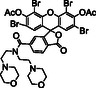	Lysosomes	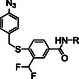
Zhou et al. [106]	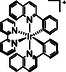	ER	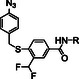

#### 3.4.4 Affinity Ligand‐Directed Photocatalytic Proximity Labeling

Ligand‐directed photocatalytic proximity labeling represents a powerful strategy for achieving target‐selective protein labeling by conjugating photocatalysts to affinity ligands. An early pioneering example of ligand‐directed photocatalysis in cellular contexts was reported by us in 2015, when we developed gefitinib‐Ru(bpy)_3_ conjugates targeting the intracellular Tyr kinase domain of EGFR [116]. Upon visible light irradiation, the ligand‐conjugated Ru(bpy)_3_ generates ^1^O_2_ that can oxidize target proteins. In the presence of labeling reagents such as *N*′‐acyl‐*N*,*N*‐dimethyl phenylenediamine, the system enables tyrosine labeling while preventing non‐specific protein oxidation. This work demonstrated the feasibility of controlling radical‐based labeling reactions with small‐molecule photocatalysts; however, the limited cell membrane permeability of Ru(bpy)_3_ complexes left room for improvement in broader intracellular applications.

In a significant evolution toward intracellular applications, Trowbridge and colleagues in 2022 transformed the μMap platform—originally developed for antibody‐based cell surface labeling—into an intracellular small‐molecule target identification tool through critical photocatalyst redesign [45]. The original first‐generation μMap catalyst, while highly effective for cell surface applications, was cell‐impermeable due to its neutral net charge and two carboxylic acid residues. Recognizing that intracellular target identification required membrane‐permeable catalysts, the team systematically optimized the photocatalyst structure while maintaining the essential photophysical properties for DET. Critically, the catalyst design retained the dF‐CF_3_‐phenyl pyridine moiety and 4,4‐dialkyl bipyridine ligand essential for achieving the requisite triplet energy for efficient diazirine activation. For synthetic accessibility and conjugation to diverse small molecules, a distal carboxylic acid was incorporated onto the 4,4‐dimethylbipyridine ligand, enabling facile amide coupling on gram scale while preserving cell permeability.

The μMap platform has been extended to peptide‐based probes through SPPS compatibility. Morgan and colleagues used HIV‐1 TAT peptide conjugates to capture temporal trafficking snapshots: 2‐min labeling identified cell‐surface proteoglycans and nuclear import factors, while 1‐hour labeling enriched nuclear proteins (82% of hits) [117].

Knutson and colleagues integrated μMap technology into targeted protein degradation (TPD) workflows through a dual‐platform strategy combining immunophotoproximity labeling (μMapX) with ligand‐directed μMap TargetID [118]. This approach addresses the fundamental “hit prioritization bottleneck” in phenotypic drug discovery, where dozens to hundreds of active compounds lack information about molecular targets or mechanisms. μMapX employs Sn‐chlorin photocatalyst‐conjugated antibodies to rapidly generate drug‐induced interactomic fingerprints of endogenous target proteins, enabling immediate triage of hit compounds based on their mechanistic signatures without requiring analog synthesis. For prioritized candidates, μMap TargetID utilizes Irphotocatalyst‐conjugated versions of the compounds to identify direct small‐molecule‐protein binding partners, providing orthogonal mechanistic validation.

#### 3.4.5 Tag Protein‐Mediated µMap

To overcome limitations in cell permeability and enable systematic profiling of intracellular organelles and biomolecular condensates, researchers developed genetically‐encoded µMap platforms utilizing protein tags that recruit photocatalysts into living cells. This strategy combines the spatial precision of photocatalytic proximity labeling with the targeting flexibility of genetic fusion proteins, providing unprecedented access to dynamic intracellular structures.

In 2024, Pan and colleagues established the HaloMap platform for mapping stress granule (SG) dynamics in living cells [119]. The approach exploits the HaloTag self‐labeling protein tag, which forms a covalent bond with chloroalkane ligands, enabling recruitment of cell‐permeable iridium photocatalysts (Ir‐PEG_4_‐hexyl‐Cl) to genetically‐tagged proteins of interest. This platform successfully identified ∼700 proteins with dynamic recruitment patterns during SG formation/disassembly and revealed HECT E3 ligases as key regulators of SG clearance through K63‐linked polyubiquitination.

Complementing HaloTag‐based approaches, split‐intein chemistry provides an alternative strategy for site‐specific photocatalyst incorporation into endogenous proteins. Applying this methodology to the oncogenic transcription factor c‐Myc, researchers utilized ultrafast split‐intein splicing to conjugate iridium photocatalysts directly to c‐Myc in living cells [120]. Following transfection with c‐Myc‐CfaN constructs and subsequent CfaC‐Ir splicing, 1‐min blue light irradiation in the presence of biotin‐diazirine probes enabled proximity labeling of the nuclear c‐Myc interactome. Mass spectrometry analysis successfully identified known c‐Myc co‐regulatory proteins among the most significantly enriched hits, validating the approach for profiling cancer‐relevant transcription factor complexes.

Addressing a critical limitation of first‐generation intracellular iridium photocatalysts—off‐target mitochondrial localization driven by cationic charge—Knutson and colleagues systematically redesigned the catalyst scaffold to enable HaloTag‐based intracellular µMap [46]. Initial screening of alternative photocatalysts, including homoleptic Ir(dFppy)_3_, revealed that only Ir(dFppy)_3_ displayed robust photolabeling activity in vitro, despite both possessing high triplet energy states (>60 kcal/mol) necessary for carbene generation. To eliminate this off‐target localization, the authors developed a intracellular µMap version 2(v2) catalyst based on an Ir(dFppy)_3_ scaffold. Chloroalkane penetration assay (CAPA) confirmed cell permeability (∼92% HaloTag incorporation at 10 µM for 16 h), and critically, immunofluorescence analysis demonstrated complete elimination of mitochondrial labeling. Comparative proteomics revealed ∼10‐fold reduction in off‐target protein labeling with a stochastic rather than organelle‐specific distribution, and cell viability assays showed significantly reduced cytotoxicity even at extended exposures.

#### 3.4.6 HaloTag‐Based Intracellular Proximity Labeling Methods Using Metal‐Free Catalysts

While Ir‐based photocatalysts have demonstrated exceptional performance in photocatalytic proximity labeling, concerns regarding material sustainability, cytotoxicity, and mitochondrial accumulation have motivated the development of organic photocatalyst alternatives. These metal‐free systems offer improved biocompatibility and expanded opportunities for intracellular applications.

We performed proximity labeling using acriflavine, a cell‐membrane‐permeable organic photocatalyst, for iPPL [28]. By conjugating acriflavine to HaloTag fusion proteins and employing MAUra as the radical labeling reagent, we achieved specific proximity labeling of histone H2B‐associated proteins in living HEK293 FT cells. Quantitative proteomics identified 33 proteins with significant light‐dependent enrichment, predominantly nuclear proteins including chromosomal components and RNA‐binding proteins. Gene ontology analysis revealed enrichment of ribonucleoprotein complexes, nucleosomes, and spliceosomal complexes. Comparative analysis with BioID and APEX revealed that iPPL exhibits a smaller labeling radius (∼6 nm), enabling more focused protein–protein interaction profiling. Extending this platform to histone H3, we demonstrated dynamic analysis of chromatin‐binding proteins through systematic optimization of acriflavine/MAUra combinations [121]. Identification of labeled amino acids in the histone H3‐interacting protein EZH2 (histone lysine N‐methyltransferase) confirmed that iPPL labels residues within a few nanometers of the target protein, validating its restricted labeling radius for high‐resolution interactome profiling.

Leveraging ^1^O_2_‐based chemistry for intracellular proximity labeling, Becker and colleagues developed POCA (Photosensitizer‐dependent Oxidation and Capture by Amine), employing the cell‐permeable rhodamine derivative JF_570_ conjugated to HaloTag ligands [52].

To enable intracellular applications of the MultiMap platform, Lin and colleagues engineered full‐length EGFR constructs bearing an extracellular N‐terminal Flag‐tag and an intracellular C‐terminal HaloTag, developing complementary strategies termed eMultiMap and iMultiMap [122]. Eosin Y was anchored extracellularly via anti‐Flag antibody conjugation (ECD ecto‐tag) or intracellularly through EY‐HaloTag ligand binding (ICD ecto‐tag), enabling simultaneous interrogation of EGFR microenvironments on both sides of the plasma membrane. Time‐resolved profiling following EGF stimulation (5–60 min) revealed dynamic proximity changes for over 300 proteins, with temporally distinct interactome networks emerging at different signaling stages.

Addressing limitations of metal‐based catalysts while maintaining high‐resolution carbene‐based labeling, Crocker and colleagues discovered deazaflavin as a biocompatible organic photocatalyst capable of diazirine activation via triplet EnT [48]. Deazaflavin (triplet energy *E*_T_ = 59.5 kcal/mol) achieves complete conversion of diazirine probes in phosphate‐buffered saline upon 450 nm irradiation, comparable to iridium catalysts but with superior biocompatibility. Cell viability assays demonstrated >85% viability even at 100 µM deazaflavin after 24‐hour incubation, contrasting sharply with cell‐permeable Ir catalysts that reduced viability to <70% at concentrations as low as 0.8 µM. Phototoxicity studies further revealed minimal cytotoxicity (>80% viability after 5 min blue light irradiation at 10 µM), attributed to competitive electron transfer from intracellular glutathione (GSH) to the deazaflavin triplet state, which mitigates ^1^O_2_‐mediated damage despite efficient oxygen sensitization (Φ_Δ_ = 62%).

Mechanistic investigations via transient absorption spectroscopy revealed that deazaflavin undergoes triplet EnT to diazirines (quantum efficiency values for triplet EnT, Φ_ET_ = 58%) and aryl azides (Φ_ET_ = 75%), while also being capable of SET to tyramide probes (quantum yield of electron transfer, Φ_eT_ = 36%). This multifaceted reactivity—termed deazaflavin‐diazirine EnT labeling (DarT labeling).

Together, these organic photocatalyst platforms significantly expand the photocatalytic proximity labeling toolkit beyond metal‐based systems, addressing critical limitations in cytotoxicity, permeability, and temporal resolution for diverse biological applications (Table 8).

**TABLE 8:** Metal‐free photocatalysts used for intracellular proximity labeling.

References	Catalyst structure	Target	Labeling reagent
Tsushima et al. [28], Miura et al. [121]	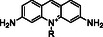	HaloTag‐H2B, ‐H3	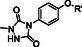
Becker et al. [52]	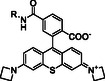	Halo‐POCA	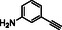
Lin et al. [122]	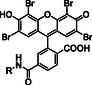	EGFR (Flag‐tag/HaloTag constructs); EY anchored via anti‐Flag–EY (ECD) or EY–HTL (ICD)	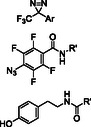
Crocker et al. [48]	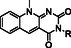	HaloTag–mGluR2	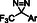

#### 3.4.7 Genetically Encoded Photocatalytic Proximity Labeling Systems

While small‐molecule photocatalysts have proven powerful for proximity labeling, they face inherent limitations, including non‐specific binding to cellular structures, uncontrolled accumulation in specific organelles, and the requirement for chemical conjugation strategies. Genetically encoded photosensitizers overcome these challenges by enabling direct fusion to any protein of interest, ensuring precise spatial specificity without concerns about off‐target localization or non‐specific interactions. This genetic approach offers particular advantages for cell biologists: target proteins can be readily changed through simple transfection of different constructs.

The pioneering work in genetically encoded photoproximity labeling utilized miniSOG, a 106‐amino acid flavin‐binding domain from *Arabidopsis thaliana* phototropin 2. Zhai and colleagues developed photoactivation‐dependent proximity (PDPL) labeling by fusing miniSOG to proteins of interest [59]. Upon blue light illumination (460 nm), the flavin mononucleotide (FMN) cofactor generates ^1^O_2_, enabling oxidation of proximal histidine residues. These oxidized histidines react with aniline‐based probes to form covalent adducts detectable by mass spectrometry. The small size of miniSOG (12 kDa)—less than half that of APEX2 (27 kDa) or TurboID (35 kDa)—minimizes potential steric interference with native protein function.

Zheng and colleagues developed ^1^O_2_‐induced protein labeling and identification (RinID), a refined approach for subcellular proteome mapping [123]. RinID capitalizes on miniSOG‐mediated photo‐oxidation where ^1^O_2_ oxidizes proximal proteins, creating electrophilic intermediates that are subsequently intercepted by nucleophilic probes. Through systematic screening, biotin‐conjugated aniline and propargyl amine emerged as optimal reagents, providing superior labeling efficiency compared to other nucleophiles. The method achieves minute‐level turn‐on kinetics while maintaining exceptional spatial specificity across multiple subcellular compartments.

Beyond protein proximity labeling, the versatility of miniSOG‐generated ^1^O_2_ has extended to nucleic acid applications. Notably, chromophore‐assisted proximity sequencing (CAP‐seq) leverages miniSOG to oxidize guanine residues in RNA, which are subsequently captured by amine probes for transcriptome mapping [124]. These RNA labeling methods demonstrate the broad utility of ^1^O_2_‐based proximity labeling beyond the proteome, though with distinct probe preferences and reaction conditions optimized for nucleotide versus amino acid modifications.

Hananya and colleagues developed a genetically encoded photoproximity labeling approach using *Arabidopsis thaliana* phototropin LOV domain (termed SOPP, LOV*) [125]. Through systematic screening of various flavin‐binding protein variants, including miniSOG fused to the N‐terminus of histone H2B, they evaluated proximity labeling efficiency using biotin‐tyramide and identified LOV* as the most efficient photocatalyst. This 12 kDa protein tag, when fused to proteins of interest, enables proximity‐dependent labeling through ^1^O_2_‐mediated activation of biotin‐tyramide probes, achieving spatial resolution of approximately 10 nm—significantly tighter than enzyme‐based approaches like BioID or APEX2.

While miniSOG‐ and LOV‐based photoproximity labeling systems are fully genetically encoded—relying on endogenous cellular flavins as intrinsic cofactors for ^1^O_2_ generation—an alternative strategy has emerged in which genetically encoded proteins are designed to bind exogenously supplied synthetic photocatalysts. This hybrid approach preserves genetic targetability while enabling access to photochemical properties unattainable with native chromophores, such as long‐wavelength excitation and enhanced tissue penetration. Within this framework, Ren and colleagues developed FLAPP (Fluorogen‐activating protein Light‐Activated Proximity Profiling), which employs the engineered fluorogen‐activating protein dL5** paired with iodinated malachite green (MG‐HI) [56]. This system operates through a fundamentally different mechanism: the FAP binding restricts rotation of the malachite green derivative, activating both fluorescence and ^1^O_2_ generation in a wash‐free manner. Critically, FLAPP enables red light activation (660 nm), overcoming the tissue penetration limitations of blue light‐dependent systems.

While direct photocatalytic proximity labeling methods like FLAPP utilize photosensitizers to generate ^1^O_2_ for direct protein labeling, alternative approaches have emerged that employ light‐controlled activation of conventional proximity labeling enzymes. Qu and colleagues developed a photoactivated proximity labeling system using SOPP3 (^1^O_2_ photosensitizing protein‐3), a LOV‐family protein that generates ^1^O_2_ under blue light illumination [126]. In this system, SOPP3‐generated ^1^O_2_ is converted to hydrogen peroxide by endogenous superoxide dismutase (SOD), which then activates APEX2 for proximity labeling without requiring exogenous H_2_O_2_. This approach achieves remarkable temporal resolution (5 s) while avoiding the cytotoxicity associated with exogenous H_2_O_2_ addition. The method demonstrated high spatial specificity at various subcellular locations, including ER‐mitochondria contact sites and cell–cell interfaces, with the chimeric APEX2‐SOPP3 fusion protein providing a versatile single‐component system for targeted proximity labeling under mild blue light illumination (50 mW/cm^2^).

Beyond direct photocatalytic approaches, optogenetic control has been integrated into proximity labeling enzymes to achieve light‐regulated activity. Lee and colleagues engineered LOV‐Turbo by incorporating the light‐sensitive LOV domain into TurboID through structure‐guided design and directed evolution, enabling rapid and reversible control of labeling activity with low‐power blue light [127]. This system achieves 30‐min temporal resolution and dramatically reduces background in biotin‐rich environments such as neurons, enabling pulse‐chase experiments to track protein trafficking between ER, nuclear, and mitochondrial compartments under cellular stress. Separately, Shafraz and colleagues developed Light‐Activated BioID (LAB), which fuses the two halves of split‐TurboID to the photodimeric proteins CRY2 and CIB1 [128]. Upon blue light illumination, CRY2 and CIB1 rapidly dimerize within 300 ms to reconstitute the split‐TurboID enzyme, initiating proximity labeling with precise temporal control. LAB demonstrated superior accuracy compared to conventional TurboID for mapping E‐cadherin interactions at the cell membrane, with significantly reduced false positives due to its on‐demand activation capability.

### 3.5 Proximity Labeling Ex Vivo

Ex vivo photocatalytic labeling is applied to primary cell preparations and tissue sections that retain aspects of native organization. Typical samples include tumor‐infiltrating lymphocytes, splenocytes, draining lymph node cells, and tissue slices, including formalin‐fixed paraffin‐embedded (FFPE) blocks. This regime preserves cell–cell contacts and tissue microenvironments better than lysates, while remaining experimentally accessible for cytometry, imaging and proteomics.

**FIGURE 15:**
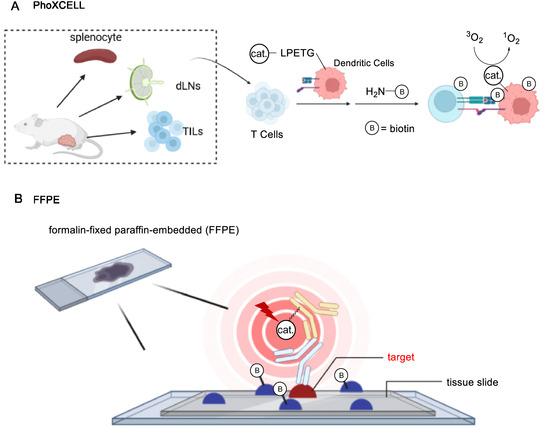
Ex vivo photocatalytic labeling: (A) PhoXCELL and CAT‐Cell for DC‐T and tumor‐T cell contacts; (B) μMap‐FFPE for receptor‐proximal proteomes in FFPE sections.

These applications have been enabled by tailored photocatalytic chemistries optimized for ex vivo conditions: ^1^O_2_ generation coupled with aliphatic amine probes for primary cell labeling, quinone methide (QM) probes for capturing cell–cell interactions in mixed populations, and µMap‐FFPE employing aniline probes specifically tuned for crosslinked tissue sections. These complementary approaches balance reactivity with tissue penetration and preservation of native structures, enabling proximity mapping in clinically relevant samples (Figure 15).

PhoXCELL (photocatalytic proximity cell labeling) is a representative example for recording antigen‐specific interactions between dendritic cells (DCs) and T cells derived from tumors [129]. In this system, immature DCs are primed with tumor lysate and labeled with a dibromofluorescein‐based photosensitizer and aliphatic amine probe. Tumor‐infiltrating lymphocytes (TILs) are isolated from the same tumor and co‐cultured with the primed DCs to allow antigen‐dependent contact. Upon visible‐light irradiation in the presence of a biotin reporter, the photosensitizer generates ^1^O_2_ at the DC‐T cell interface, leading to proximity‐dependent biotinylation of T cells in contact with antigen‐presenting DCs.

CAT‐Cell uses a different photocatalytic chemistry to label T cells that engage in direct contact with tumor cells [130]. Tumor or antigen‐presenting cells are modified on their surface with an NHS‐ester‐linked Ir photocatalyst (NHS‐Ir) and loaded with cognate or control peptide. When these bait cells are mixed with T cells in the presence of a caged quinone‐methide–biotin probe such as PAB‐QM‐Bio and illuminated with blue light, the surface‐bound Ir catalyst decages the probe to release a short‐lived quinone methide intermediate. In co‐cultures of splenocytes with OVA‐primed MC38 tumor cells, CAT‐Cell selectively biotinylates OVA‐specific OT‐I CD8^+^ T cells in an antigen‐dependent manner, while non‐cognate peptide conditions show minimal labeling. Applied ex vivo to tumor‐infiltrating lymphocytes from syngeneic MC38 tumor models, CAT‐Cell reveals reduced tumor‐specific biotinylation of CD8^+^ T cells in MC38‐B2M knockout tumors, consistent with impaired MHC‐I antigen presentation.

μMap‐FFPE adapts proximity labeling to FFPE tissue sections, which are abundant in clinical archives but difficult to handle with standard photochemical probes [131]. Initial attempts to apply μMap‐Blue showed that FFPE processing introduces endogenous photosensitizers that activate diazirine probes under blue light, leading to substantial background even in the absence of the Ir photocatalyst. In addition, conventional high‐temperature de‐cross‐linking (95ºC) promotes thermal activation of residual biotin‐azide probe, generating nitrenes that label proteins nonspecifically and destroy spatial information. To address these problems, the μMap‐Red system uses a Sn‐chlorin photosensitizer that absorbs red light, together with a thermally stable biotin–aniline probe. Under optimized extraction and de‐cross‐linking conditions, labeling in FFPE human tonsil tissue requires both the Sn catalyst and red‐light irradiation, restoring spatiotemporal control.

Beyond cell‐contact mapping and FFPE‐compatible receptor‐proximal profiling, ex vivo photocatalytic proximity labeling has also been extended to pathological protein deposits in disease tissue. Feng and colleagues developed Amyloid‐ID, in which a Thioflavin T‐derived photocatalyst, P9, was applied to Alzheimer's disease mouse brain sections to achieve spatially confined in situ labeling of amyloid deposits, followed by direct enrichment and LC–MS/MS analysis from labeled tissue sections. This workflow identified 357 deposit‐associated proteins, including many neurodegeneration‐related and metabolism‐related factors, and notably recovered MAPT/tau, which had been absent from some previous amyloid proteomic datasets. Comparative amyloidomic analysis across three AD mouse models further revealed a broadly shared protein signature within amyloid deposits, with prominent enrichment of mitochondria‐associated proteins, suggesting a common mitochondrial entanglement pattern in these pathological assemblies. Collectively, this study illustrates how small‐molecule photocatalytic labeling can preserve tissue‐context information while enabling proteomic dissection of disease‐associated aggregates at ex vivo resolution [132].

These ex vivo applications show that photocatalytic labeling can be used in settings that retain tissue architecture and immune microenvironments. They provide functional readouts, such as the correlation between labeling and cytokine secretion or checkpoint expression, and they extend proximity proteomics to FFPE samples. At the same time, they must contend with limited light penetration, diffusion barriers in tissues and a balance between permeabilization or de‐cross‐linking and preservation of labeling specificity. Compared with enzyme‐based methods, photocatalytic ex vivo platforms offer rapid temporal control and flexible targeting via antibodies or sensitizers, but are more sensitive to photochemical artifacts and optical inhomogeneity. In practice, ex vivo systems serve as a useful intermediate for optimizing conditions and readouts under realistic tissue‐level constraints before moving into fully in vivo experiments.

### 3.6 Proximity Labeling In Vivo

**FIGURE 16:**
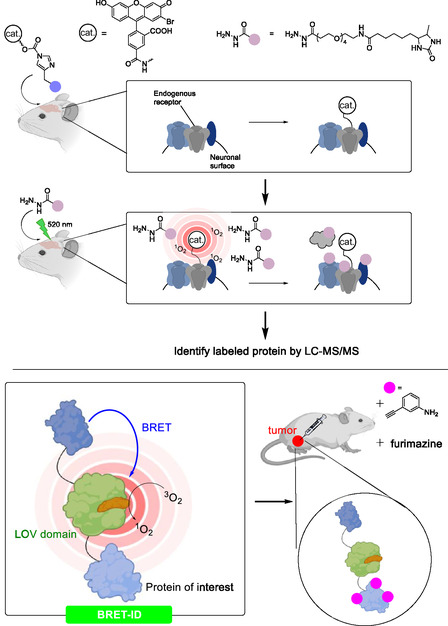
Activation modes and spatial scales of in vivo photocatalytic labeling: PhoxID and LAP/BRET‐ID (bioluminescence at subcellular structures).

In vivo photocatalytic labeling applies proximity mapping directly in living animals, representing the highest level of biological complexity. These approaches deliver photocatalysts, nanozymes, or bioluminescent fusion proteins to target organs, where labeling is triggered by external light, ultrasound, or bioluminescence to map receptor microenvironments and cell–cell interactions under physiological conditions (Figure 16).

PhoxID (photooxidation‐driven proteome identification) demonstrates that receptor microenvironments in genetically intact mouse brain can be mapped without genetic manipulation [133]. Ligand‐directed acyl imidazole reagents deliver 2‐monobromofluorescein photosensitizers to either AMPA‐type glutamate receptors (MBF‐AI‐PFQX) or GABAA receptors (MBF‐AI‐FMZ) via intracerebroventricular injection. Following 24–28 h equilibration for receptor targeting through cerebrospinal fluid circulation, a hydrazide‐desthiobiotin reporter (Hyd‐PEG4‐dBt) is microinjected into specific brain regions, and green light (520 nm, 0.16 W cm^−2^) is delivered through implanted optical fibers for 10 min. The photosensitizer generates ^1^O_2_ locally, oxidizing proximal proteins for covalent reporter attachment.

Spatial analysis revealed labeling predominantly within 1 mm of the injection/irradiation site, consistent with green light attenuation in brain tissue, enabling precise anatomical targeting. Temporal profiling across postnatal development stages (postnatal day 8 to week 5) in cerebellum revealed dynamic AMPAR microenvironment remodeling, with developmental stage‐specific enrichment of synaptic organizers and adhesion molecules, highlighting PhoxID's capability for hypothesis‐free discovery of transient proteome changes during circuit maturation in intact brain tissue.

SeeID (silicon‐rhodamine‐enabled identification) is a representative example of such a genetically encoded platform and couples red‐light imaging and photocatalytic labeling in a single HaloTag‐based module [134]. Silicon rhodamine (SiR) is covalently attached to HaloTag fusion proteins; binding of SiR to HaloTag switches on fluorescence and enables ^1^O_2_ generation under 660 nm light. The same Halo‐SiR module supports organelle‐resolved proximity labeling in cultured cells, but its most distinctive feature in the present context is its application in vivo. In live mice, Halo‐tagged PDGFR‐TM protein (Halo‐TM) is introduced into the cerebral cortex using an AAV virus‐carrying system, SiR‐HaloTag ligand and a biotinylated aniline probe are locally delivered, and 660 nm LED irradiation (typically 50 mW cm^−2^ for 30 min) is applied through the skull. Under these conditions, SeeID produces SiR‐dependent labeling across the mouse cortex with an effective depth of about 2 mm. Proteomic analysis of cortical lysates identifies approximately 60 enriched proteins (SiR‐HaloTag ligand/DMSO ratio >1.5, *p* < 0.05), a majority of which are annotated as membrane‐, vesicle‐ or extracellular‐matrix‐associated, including synaptic receptors and adhesion molecules at the plasma membrane. These results show that red light‐driven SeeID can access membrane‐proximal proteomes in intact brain tissue and extend photocatalytic proximity labeling to deep regions that are largely inaccessible to blue‐light systems.

Other in vivo‐compatible photocatalytic platforms emphasize different biological questions. Li and colleagues repurposed proximity‐labeling chemistry to amplify antigen‐induced receptor clustering directly in tumor‐bearing mice [135]. Using porous coordination network (PCN) nanoparticles that contain porphyrin and zirconium as catalysts, they first install high densities of fluorescein tags around selected antigens in tumors through photocatalytic reactions with tyramide or amine probes under the control of red light or ultrasound. A fluorescein‐binding bispecific T cell engager then accumulates selectively at the tumor site in vivo. This local clustering enhances T cell activation and cytotoxicity in syngeneic mouse tumor models. The amplified clustering leads to rapid eradication of treated tumors. It also induces epitope spreading and systemic immunity against untreated distal lesions, together with long‐term immune memory upon tumor rechallenge. These results illustrate that photocatalytic labeling can be used not only to observe receptor microenvironments but also to manipulate antigen‐dependent signaling in vivo.

Sun and colleagues developed the BRET‐ID platform to avoid external light and achieve precise spatial proteomics in living systems [60]. A NanoLuc‐LOV domain fusion generates blue light in situ via bioluminescence resonance EnT. This light activates ^1^O_2_ ‐based labeling only at the site of the tagged protein. BRET‐ID has been applied not only in cultured cells but also in tumor xenografts. In these in vivo tumor models, BRET‐ID records G3BP1‐centered SG proteomes under oxidative stress. Proteomic analysis reveals new SG components, including the mTORC2 subunit RICTOR, in an in vivo tumor context. Building on this bioluminescence‐triggered concept, Wang and colleagues recently reported a bioluminescence‐activated proximity‐labeling platform for spatial multi‐omics [136]. Their system fuses NanoLuc luciferase with the miniSOG photocatalyst, enabling BRET‐driven ^1^O_2_ generation for proximity labeling. Through yeast display‐directed evolution, they optimized the fusion protein for enhanced activity, creating the NanoLuc‐miniSOG variant with improved miniSOG (SOPP2I34V/I55V) and optimized linker sequences that demonstrates high spatial specificity across multiple subcellular compartments. This bioluminescence‐based approach provides a versatile toolkit for spatial multi‐omics without the tissue penetration limitations of light‐activated systems.

Taken together, these in vivo photocatalytic labeling strategies extend proximity labeling to intact organs and whole animals, allowing receptor microenvironments, organelle‐ and membrane‐proximal proteomes, and antigen‐dependent cell–cell interactions to be characterized under physiological conditions. Their strengths include high spatiotemporal resolution, the ability to capture transient or developmentally regulated changes in local proteomes, and, in some cases, the possibility to couple microenvironment mapping with functional modulation of immune responses. At the same time, they face challenges such as efficient and selective delivery of photocatalysts or fusion constructs in vivo, control of phototoxicity and background arising from endogenous chromophores, and constraints imposed by light penetration or the availability of suitable bioluminescent triggers. As these limitations are addressed, in vivo photocatalytic labeling is likely to become a powerful complement to enzyme‐based proximity labeling and classical imaging for dissecting protein networks in their native physiological context.

### 3.7 Performance Considerations in Biological Systems

The preceding sections have documented the broad applicability of photocatalytic proximity labeling across biological systems of increasing complexity. However, a systematic evaluation of how different photocatalytic approaches perform under realistic biological conditions—and how they compare with established enzyme‐based platforms—remains essential for guiding method selection and experimental design. Three interrelated dimensions merit particular attention: signal‐to‐noise characteristics, perturbation of native biology, and the consequences of catalyst localization strategies. This section addresses each in turn and concludes with a practical framework for navigating the trade‐offs inherent in photocatalytic proximity labeling.

#### 3.7.1 Signal‐to‐Noise and Background Control

A defining operational advantage of photocatalytic proximity labeling is light‐gated activation: reactive intermediates are generated only during defined irradiation windows, and in the absence of light, photocatalysts remain chemically inert. This feature provides an inherently clean negative control—omitting illumination eliminates labeling—that is more stringent than the substrate‐addition controls used with BioID/TurboID, where promiscuous biotin ligases can exhibit residual (‘leaky’) activity even without exogenous biotin supplementation [61, 62]. In the iPPL system, quantitative proteomics confirmed that enrichment was strictly light‐dependent, with negligible labeling in dark controls [28, 121]. Similarly, PhoTag and PhoXCELL demonstrated greater than twofold signal reduction in the absence of either photocatalyst or light [29, 129].

Despite this intrinsic advantage, photocatalytic systems are subject to several sources of background labeling that must be managed experimentally. First, dark‐state background can arise from non‐specific interactions between the photocatalyst and cellular components. Cationic iridium complexes, for example, accumulate preferentially in mitochondria due to the negative mitochondrial membrane potential (∼−180 mV), generating organelle‐specific off‐target labeling that contaminates datasets with mitochondrial proteins even when the intended target resides elsewhere [46]. This problem has been addressed by redesigning the catalyst scaffold: the µMap v2 Ir(dFppy)_3_ catalyst eliminated mitochondrial accumulation and reduced off‐target labeling approximately 10‐fold compared with the first‐generation cationic catalyst [46].

Second, light‐dependent background can arise from the activation of endogenous photosensitizers. Blue light (450 nm) excites cellular flavins and porphyrins to generate ROS, causing non‐specific oxidation that confounds proximity‐dependent signals [11]. This problem is particularly acute in FFPE tissue sections, where formaldehyde‐induced autofluorescent species further activate diazirine probes under blue illumination [131]. The development of red‐ and NIR‐responsive catalysts has largely circumvented this limitation: µMap‐Red (660 nm, Sn‐chlorin) [23], Os‐based systems (660 nm) [94], and CAT‐Tissue (650–700 nm, chlorin e6) [54] all operate at wavelengths beyond the absorption of endogenous chromophores, restoring the signal specificity that is compromised under blue‐light conditions.

Third, probe‐derived background can occur through thermal or non‐specific activation of labeling reagents. The thermally labile biotin‐azide probe used in initial µMap‐FFPE attempts generated nitrenes upon high‐temperature de‐cross‐linking, producing light‐independent background. Replacing this probe with a thermally stable biotin‐aniline derivative eliminated the artifact [131].

Relative to enzyme‐based methods, photocatalytic systems typically yield smaller but more spatially defined datasets. APEX‐catalyzed labeling (labeling radius ∼200–269 nm [63, 64]) generates large proteome lists that include many proteins beyond direct interaction partners, whereas µMap (effective FWHM ∼54 nm with antibody tether, intrinsic carbene diffusion ∼4 nm [64]) and iPPL (∼6 nm [28]) produce compact interactomes enriched for direct neighbors. This trade‐off between coverage and precision should be considered when selecting a platform: broad organelle surveys may benefit from the amplification of enzymatic methods, while high‐resolution microenvironment mapping favors spatially confined photocatalytic approaches. The chemical basis for these differences—intermediate lifetime, diffusion distance, and spatial confinement—is discussed in Section 2.4 and summarized in Table 1.

These quantitative differences across platforms are summarized in Table 2, which compares enzyme‐based and photocatalytic proximity labeling methods with respect to labeling radius, temporal resolution, reactive intermediate, activation trigger, and catalyst localization strategy.

#### 3.7.2 Perturbation of Biological Systems

While Table 9 summarizes the key chemical distinctions between SET and EnT pathways, all proximity labeling methods perturb the biological system under study to some degree.

**TABLE 9:** Mechanistic comparison of photocatalytic protein modification strategies. This table compares the chemical properties and reaction characteristics of SET, EnT (TTET/^1^O_2_), and EnT (DET) pathways. For comparison with enzyme‐based proximity labeling methods and evaluation of biological performance including signal‐to‐noise, perturbation, and cytocompatibility, see Section 3.7 and Table 2.

Category	SET	EnT (TTET/^1^O_2_)	EnT (DET)
Activation mode	Light irradiation(Visible–NIR light)		
Reactive species	Radicals	^1^O_2_, carbene, nitrene	Carbene, nitrene
Primary mechanism	SET from/to excited photocatalyst	EnT to ^3^O_2_	EnT to diazirine/azide (orbital overlap required)
Oxygen dependence	Not required for activation; O_2_ may participate in catalyst turnove	Required	Not required
Catalyst localization strategies	Ligand/antibody/genetic fusion		
Biological perturbation	Redox imbalance (oxidation/reduction)	ROS‐induced oxidative stress	Minimal–moderate
Key advantages	Broad residue scope; tunability via redox potential	Orthogonal residue selectivity (His); wavelength flexibility	Highest spatial confinement; no ROS; no O_2_ dependence
Key chemical limitations	Competing overoxidation	ROS damage; oxygen dependence	Requires high triplet energy catalyst

##### 3.7.2.1 ROS‐Mediated Perturbation in EnT/^1^O_2_ Systems

Photocatalysts that generate singlet oxygen (^1^O_2_) through TTET inevitably produce a potent oxidant that can damage proteins, lipids, and nucleic acids beyond the intended labeling targets. The parent molecule miniSOG was originally developed for chromophore‐assisted light inactivation (CALI) and cell ablation [57], underscoring its inherent phototoxicity at high light doses. The severity of this perturbation depends on the balance between ^1^O_2_ generation rate, quenching by endogenous antioxidants, and the irradiation dose. In practice, most ^1^O_2_‐based proximity labeling protocols employ short irradiation times (typically 1–10 min) that are well below the threshold for gross cytotoxicity, though subcellular oxidative stress at the labeling site cannot be excluded.

A further practical constraint of ^1^O_2_‐based systems is their obligate oxygen dependence (see Section 2.3.2 for the mechanistic basis): in hypoxic microenvironments such as tumor cores, ischemic tissues, or densely packed three‐dimensional organoids, reduced O_2_ tension may attenuate ^1^O_2_ production and compromise labeling coverage. DET‐based systems are operationally independent of local O_2_ concentration [44].

By contrast, DET‐based systems such as µMap generate short‐lived carbene or nitrene intermediates without producing ROS [44]. Because diazirines are activated through direct triplet EnT from the photocatalyst rather than through oxygen‐dependent pathways, the DET mechanism avoids oxidative perturbation entirely. This distinction is particularly relevant for studying redox‐sensitive processes, where even modest ROS bursts could alter the interactome under investigation. The recently reported deazaflavin photocatalyst represents an instructive comparison: despite exhibiting efficient ^1^O_2_ generation (Φ_Δ = 62%), competitive electron transfer from intracellular glutathione (GSH) to the deazaflavin triplet state effectively quenches ^1^O_2_‐mediated damage, yielding cell viability >85% at 100 µM after 24‐hour incubation and >80% viability after 5 min blue‐light irradiation at 10 µM [48]. In contrast, cell‐permeable Ir catalysts reduced viability to <70% at concentrations as low as 0.8 µM [48].

##### 3.7.2.2 Redox Perturbation in SET Systems

SET‐mediated photocatalysis generates radical intermediates through direct electron transfer between the excited photocatalyst and either protein residues or labeling reagents. In this process, strongly oxidizing excited‐state species such as Ir(III)* or Ru(II)* can oxidize amino acid side chains non‐specifically, leading to overoxidation and unintended crosslinking [13]. Moreover, the catalytic cycle in SET systems may consume cellular reductants—GSH (1–10 mM in the cytosol [96, 97]) and NADH that serve not only as quenchers that limit radical diffusion but also as sacrificial electron donors that maintain the photocatalytic cycle [23]. Sustained SET photocatalysis could therefore shift the intracellular redox balance, a perturbation that is difficult to detect but potentially consequential for redox‐sensitive signaling pathways.

##### 3.7.2.3 Temporal Trade‐Offs

The choice of irradiation time represents a practical trade‐off between labeling yield and biological perturbation. The SOPP3–APEX2 hybrid system achieves sufficient labeling in as little as 5 s [126], while iPPL and µMap typically require 1–10 min [28, 44], and BioID needs 12–24 h of biotin incubation [61]. Longer irradiation increases proteome coverage but also amplifies ROS accumulation and phototoxicity. As a general principle, the minimum irradiation time that yields statistically robust enrichment should be determined empirically for each biological system. Time‐course experiments that monitor both labeling yield and cell viability—as demonstrated for deazaflavin [48] and SOPP3 [126]—provide a rational basis for this optimization.

#### 3.7.3 Catalyst Localization and Its Impact on Labeling Precision

The strategy used to position the photocatalyst at its biological target profoundly affects both the apparent labeling radius and the specificity of the resulting interactome. Four principal localization strategies have been employed, each with distinct advantages and constraints.

**Antibody conjugation** provides high target specificity via antigen recognition and requires no genetic manipulation, making it the method of choice for cell‐surface and ex vivo applications [44, 50, 77, 129]. However, the physical dimensions of the antibody assembly (IgG ∼10–15 nm; IgG + secondary antibody ∼25–30 nm) contribute substantially to the observed labeling radius. Oakley and colleagues demonstrated that the µMap carbene FWHM of 54 ± 12 nm was nearly identical to the cluster size of biotinylated secondary antibodies alone (57 ± 12 nm), indicating that the antibody tether, rather than carbene diffusion, was the dominant determinant of apparent spatial resolution [64]. Strategies to reduce this tether contribution include the use of nanobodies (VHHs, ∼2–4 nm) [77] and direct conjugation of catalysts to primary antibodies, which reduced the maximum labeling distance to approximately 11 nm [65].

**Small‐molecule ligand conjugation** minimizes tether length by attaching the photocatalyst directly to an affinity ligand. This approach is uniquely suited for intracellular target identification, as demonstrated by the second‐generation µMap catalyst conjugated to cell‐permeable drug‐like molecules [45] and by the gefitinib–Ru conjugate targeting EGFR kinase [116]. The absence of an antibody tether means that the observed labeling radius more closely reflects the true diffusion distance of the reactive intermediate. However, ligand‐directed strategies require that a suitable high‐affinity ligand exists for the protein of interest, and non‐specific binding of the ligand–catalyst conjugate to off‐target proteins can compromise specificity.

**Genetic fusion** (HaloTag, SNAP‐tag, split‐intein, or direct protein fusion) provides the most precise catalyst positioning by covalently tethering the photocatalyst or photosensitizer directly to the protein of interest. iPPL achieved a labeling radius of approximately 6 nm via acriflavine–HaloTag conjugation [28], while split‐intein splicing enabled traceless catalyst installation on endogenous histones for chromatin interactome mapping [89]. The principal limitations of genetic fusion strategies are the requirement for exogenous expression—which may cause overexpression artifacts—and the potential for the fused tag to perturb native protein function. For genetically encoded photosensitizers such as miniSOG (12 kDa) and LOV* (12 kDa) [125], the smaller size relative to APEX2 (27 kDa) or TurboID (35 kDa) partially mitigates steric concerns.

**Free‐catalyst distribution** relies on the physicochemical properties of the photocatalyst to achieve subcellular localization without targeting moieties. Lipophilic cationic dyes such as Rhodamine 123 [51], IR780 [55], and first‐generation Ir catalysts [67] accumulate in mitochondria driven by the membrane potential, while Hoechst‐conjugated photosensitizers target chromatin [111, 112]. Free‐catalyst approaches are experimentally simple and do not require genetic manipulation, but they sacrifice targeting precision: accumulation is governed by bulk physicochemical properties rather than molecular recognition, and dual localization (e.g., cationic Ir in both cytoplasm and mitochondria) can confound spatial interpretation. The µMap v2 catalyst, which replaced the first‐generation cationic Ir scaffold with a neutral Ir(dFppy)_3_ derivative, demonstrated that rational catalyst redesign can eliminate organelle‐specific off‐target accumulation [46].

#### 3.7.4 Practical Considerations for Method Selection

The choice among photocatalytic proximity labeling platforms should be guided by the biological question, the required spatial resolution, the tolerance for perturbation, and practical constraints such as the availability of genetic tools. DET‐based approaches (µMap, deazaflavin DarT) provide the highest spatial resolution and the lowest perturbation, but require specialized catalysts and, for intracellular applications, careful attention to catalyst delivery and off‐target accumulation. ^1^O_2_‐based approaches (miniSOG, POCA, CAT‐Prox series) offer genetic encodability and broad applicability across organelles, but carry inherent phototoxicity risks that must be mitigated through dose optimization. SET‐based radical approaches (iPPL, flavin‐tyramide systems) occupy an intermediate position, providing moderate spatial resolution with well‐characterized radical chemistry, though redox perturbation should be considered for sensitive biological contexts. Red‐ and NIR‐light systems [23, 47, 54, 94] address light‐penetration and background‐labeling limitations but are currently available for fewer catalyst classes. As the field continues to diversify its chemical toolkit, hybrid strategies—combining the genetic targetability of protein‐based photosensitizers with the catalytic efficiency of optimized synthetic photocatalysts [48, 126]—are likely to offer the most flexible solutions for spatially resolved proteomics across biological scales.

## 4 Conclusions and Outlook

Photocatalyst‐driven protein labeling has evolved from isolated demonstrations of light‐triggered reactivity into a broadly applicable chemical framework for encoding spatial information in biological systems. The developments summarized in this review collectively highlight a defining feature of photocatalytic approaches: the ability to translate molecular proximity into covalent chemical records through mechanisms that are governed by photophysics and reaction context rather than enzymatic turnover alone.

Table 10 provides a chronological overview of the key methodological advances discussed in this review, organized by photocatalyst class and application domain. This timeline illustrates how the field has progressed from foundational demonstrations of Ru‐catalyzed protein modification through the introduction of DET‐based high‐resolution platforms and genetically encoded photosensitizers to recent in vivo applications.

**TABLE 10:** Timeline of key advances in photocatalytic proximity labeling (up to 2025).

Year	Ru photocatlyst (SET)	μMap (DET/Carbene)	^1^O_2_ (TTET)	CAT‐Prox	Genetically encoded	Red/NIR light	Metal‐free organic
2013	Ligand‐directed Ru SET [15]						
2015	Gefitinib–Ru EGFR [116]						
2018–2019	MAUra Tyr labeling [69]BH3–Ru MCL‐1 [16]		DBF‐Hoechst nucleus [111, 112]				Flavin C‐term mod [25]
2020–2021		μMap (DET) [44]	^1^O_2_ His umpolung [43] LUX‐MS [82]	CAT‐Prox mito [67]			Met‐selective mod [26] Tyr bioconjug [71]. Rhodamine 123 mito [51]
2022	Ru‐TAP Trp‐selective [17]	2nd‐gen Ir (intracell.) [45] SARS‐CoV‐2 μMap [84] GlycoMap [86]Radius measurement [64]	PhoTag cell–cell [77]PhoXCELL DC–T [129] TPP‐DBF mito ^1^O_2_ [49]		PDPL (miniSOG) [59]	μMap‐Red Sn‐chlorin [23]	iPPL (acriflavine) [28]
2023	SET/TTET switching [18]Ru SET vs ^1^O_2_ [81]Switchable DNA cat [79].	μMap chromatin [89] μMap binding site [73]SARS‐CoV‐2 spike [85]			RinID (miniSOG) [123] LOV* photoproximity [125] LOV‐Turbo [127]	Os deep‐red (660 nm) [94]	RFT Tyr radical mech [29].
2024		Single‐site calibration [65] μMap‐Interface [88]	Global His profiling [102]	CAT‐S primary cells [103]CAT‐Cell tumor–T [130]		Red‐light diazo→carbene [47] NIR tissue fluoroalkyl [31].	MultiMap (Eosin Y) [50] iPPL histone H3 [121]
2025		μMap v2 condensates [46] HaloMap SG dynamics [119] Split‐μMap ligation [87] μMap‐FFPE tissue [131]		CAT‐Lyso lysosomes [114] CAT‐ER dynamics [106]CAT, seq RNA multi‐omics [104]	FLAPP NIR (660 nm) [56]BRET‐ID (no light) [60] NanoLuc‐miniSOG [136] SOPP3–APEX2 [126]	CAT‐Tissue deep‐red [54] PL‐NIR IR780 [55] Multiprobe red EGFR [95] SeeID in vivo (660 nm) [134]	Deazaflavin DarT [48] FP chromophore mimics [108] eMultiMap/iMultiMap [122] PhoxID mouse brain [133]

A key conceptual advance in this field is the recognition that photocatalysis enables programmable proximity chemistry. Representative examples include proximity‐dependent tyrosine labeling using Ru photocatalysts and urazole‐derived coupling reagents such as MAUra, localized SET, rapid generation of short‐lived reactive intermediates, and restricted diffusion together impose a stringent distance requirement, such that efficient labeling occurs only when the catalyst and target residue reside within a few nanometers. These and related strategies transform classical photoredox reactivity into a spatially gated chemical logic, demonstrating that proximity itself can be encoded as selectivity. The extension of these concepts to iPPL further illustrates that photocatalytic systems can operate in complex, redox‐buffered cellular environments, providing spatial resolution that complements, and in certain contexts exceeds, that of established enzyme‐based methods.

Equally important is the realization that photocatalytic protein modification is inherently context‐dependent. Studies using Ru‐based systems reveal that the same photocatalyst can function either as a localized electron transfer source or as a ^1^O_2_ generator, with the operative pathway switching between SET‐type and EnT‐type mechanisms depending on oxygen availability, catalyst localization, and reaction partners. Rather than representing an experimental complication, this mechanism of switching emerges as a powerful design principle, underscoring that photocatalytic proximity labeling should be viewed as a reaction system whose behavior arises from the interplay between photophysical properties, local chemical environment, and macromolecular architecture.

From a spatial perspective, photocatalytic strategies offer a level of tunability that is difficult to achieve with enzymatic platforms. EnT‐based approaches span a wide range of effective labeling radii, from sub‐nanometer regimes associated with carbene or nitrene intermediates to several nanometers for ^1^O_2_‐mediated oxidation, while SET‐based radical processes occupy an intermediate space defined by diffusion length and trapping kinetics. As a result, photocatalytic proximity labeling does not simply report binary interaction partners, but rather delineates chemically defined molecular neighborhoods whose boundaries depend on catalyst design, activation mode, and biological context. Interpreting such data therefore requires careful consideration of the underlying chemistry alongside structural and cellular information.

Photocatalytic proximity labeling is best viewed as complementary to enzyme‐based approaches such as APEX and BioID. While enzymatic systems provide signal amplification and ease of genetic encoding, photocatalytic methods uniquely offer light‐gated temporal control, adjustable spatial resolution, and compatibility with non‐genetic targeting strategies, including small‐molecule ligands and antibodies. Recent hybrid strategies, including bioluminescence‐driven photocatalysis and genetically encoded photosensitizers, further suggest a convergence of chemical and biological recording technologies, blurring traditional distinctions between these platforms.

Ongoing advances in photocatalyst design, particularly the development of red‐ and NIR‐responsive systems, are expected to expand the applicability of proximity labeling to thick tissues and in vivo settings. Addressing challenges related to phototoxicity, background reactivity, and catalyst delivery will be critical for broader adoption. Beyond static mapping of interactomes, photocatalytic strategies hold promise for more active applications, including spatially confined perturbation of protein networks and localized modulation of signaling pathways.

In summary, photocatalytic proximity labeling has matured into a chemically sophisticated and biologically versatile approach for probing molecular organization. By treating photocatalysis as a programmable interface between light and biology—rather than as a fixed reaction—the field is poised to move beyond descriptive mapping toward causal interrogation and control of biomolecular interactions. This perspective positions photocatalytic proximity labeling as a central component of next‐generation chemical biology, with implications that extend from fundamental studies of cellular architecture to translational applications in diagnostics and therapeutics.

## Funding

This work was supported by the Japan Science and Technology Agency (JPMJFR2005), Japan Society for the Promotion of Science (23H02099, 24K18252), and The NAGASE Science Technology Foundation.

## Conflicts of Interest

The authors declare no conflicts of interest.

## Data Availability

The data that support the findings of this study are available from the corresponding author upon reasonable request.
